# Appraisal of the Use of Proteomics Methodological Approaches and Technologies on Sheep and Goat Research and Clinical Work

**DOI:** 10.3390/ani15203050

**Published:** 2025-10-20

**Authors:** Maria V. Bourganou, Georgia A. Vaitsi, Dimitra V. Liagka, Charalambia K. Michael, Eleni I. Katsarou, Dimitris C. Chatzopoulos, Natalia G. C. Vasileiou, Elias Papadopoulos, George Th. Tsangaris, Daphne T. Lianou, Vasia S. Mavrogianni, George C. Fthenakis, Angeliki I. Katsafadou

**Affiliations:** 1 Faculty of Public and One Health, University of Thessaly, 43100 Karditsa, Greece; mbourganou@uth.gr (M.V.B.); agkatsaf@uth.gr (A.I.K.); 2 Veterinary Faculty, University of Thessaly, 43100 Karditsa, Greece; 3 Faculty of Animal Science, University of Thessaly, 41110 Larissa, Greece; 4 School of Veterinary Medicine, European University of Cyprus, Egkomi, Nicosia 2404, Cyprus; 5 Laboratory of Parasitology and Parasitic Diseases, School of Veterinary Medicine, Faculty of Health Sciences, Aristotle University of Thessaloniki, 54124 Thessaloniki, Greece; eliaspap@vet.auth.gr; 6 Proteomics Research Unit, Biomedical Research Foundation of the Academy of Athens, 11527 Athens, Greece

**Keywords:** animal science, bibliometric, bibliometric analysis, biomarkers, goat, health sciences, LC-MS/MS, mastitis, meta-research, One Health, proteome, research analysis, reproduction, research mapping, review, sheep, small ruminant, veterinary science

## Abstract

This paper describes a detailed evaluation of published works, in which proteomics methodological approaches and technologies were used to advance knowledge about small ruminants. The specific objective of this assessment was the presentation of quantitative characteristics on the content and the bibliometric details of publications on the use of proteomics methodological approaches and technologies in small ruminant work. For the search of published papers, the following topical search string was used: [sheep OR ovine OR *Ovis aries* OR goat* OR caprine OR *Capra hircus*] AND [proteom*], in the Web of Science database. In total, 481 published papers (448 original articles and 33 reviews) were evaluated individually. These originated from 56 countries, mostly from China and the United States of America, and increased gradually with time. Most (85.7%) original articles presented experimental work with animals, most often in sheep/goat production (37.4%) and reproduction (21.8%), and less often in physiology (19.0%) or diseases (17.9%). Tissues analyzed more often were milk (17.9%), blood (10.3%), and muscle (9.2%). The proteomics methodological approach and technology employed most frequently was the liquid chromatography-tandem mass spectrometry (52.9% of articles). The journal, in which papers were published most frequently, was the *Journal of Proteomics* (8.3%). There were 3689 authors in total in the published papers, with a median number of 7 per paper Median number of references was 50 per published paper. Median number of citations was eight per published paper, and median number of annual citations was 2.0 per published paper. Significant predictors for the number of annual citations were (a) for reviews, the number of references, and (b) for original articles, the number of references, the topic of study, and the animal species referred to in the articles. The Epimetre briefly reviews the contribution of proteomics in the health management of sheep and goats. Overall, the results have indicated that the use of proteomics methodological approaches and technologies in sheep and goat work has advanced our knowledge and understanding of the biology of these two animal species in a multitude of fields and topics internationally, with an increasing dissemination and applicability.

## Simple Summary

The present work focuses on the presentation of quantitative characteristics of the content and the bibliometric details of publications on the use of proteomics methodological approaches and technologies in small ruminant work. The results indicated that the use of proteomics methodological approaches and technologies in sheep and goat work has advanced our knowledge and understanding of the biology of these two animal species in a multitude of fields and topics internationally, with an increasing dissemination and applicability. Moreover, the epimetre briefly reviews the contribution of proteomics in the health management of sheep and goats. Through the study of protein expression by means of these methodologies, it has become possible to improve production practices, as well as to elucidate disease mechanisms. The use of these technologies has been reflected in higher production yields in sheep and goat farms, in improved diagnosis of diseases, and in the implementation of better control schemes against infections.

## 1. Introduction

Proteomics is defined as the large-scale study of the protein structure and function in biological systems, primarily through the comprehensive identification and quantification of proteins [1]. Through the evaluation and the assessment of the entire protein complement (‘proteome’) of a cell, tissue, or organism, proteomics methodologies will provide insights that extend beyond genomics data. In contrast to genomics methodologies, which analyze the relatively static DNA sequences, proteomics can capture the dynamic state of various biological systems and can elucidate changes in protein expression, modification, and interaction in real time, thereby providing valuable information of significant physiological relevance and clinical importance [2,3,4].

This dynamic perspective means that proteomics methodologies and analyses can offer a ‘snapshot’ of a pathological condition at a given moment [3,5]. Notably, unlike the genome, which remains relatively constant, the proteome is highly dynamic [6]. Consequently, proteomics reflects real-time cellular activities and biochemical statuses, which enable the discovery of biomarkers and molecular signatures associated with health or disease [5]. For example, changes in the proteome between healthy and ‘diseased’ tissues can be used to identify protein markers for the early diagnosis of pathological conditions or targets for their treatment, underscoring proteomics value in biomedical research [5]. Large proteomic datasets can illustrate the interdependence of cellular processes crucial for normal growth and responses to diseases [3].

In the field of veterinary and animal science research, proteomics has emerged as a powerful tool, as it can provide valuable insights into biological mechanisms related to animal growth, reproduction, health, and disease (especially in economically important livestock and poultry) [7]. Proteomics have been employed, among others, as a means to elucidate pathogenetic mechanisms of diseases, to identify novel diagnostic approaches through biomarker discovery, and to target therapeutic approaches in various diseases. The use of proteomics in small ruminant research and clinical work (sheep, goats) has not been as wide as in other animal species, even though these two species play a crucial role in global food production and in rural livelihoods. This results in a knowledge gap, as proteomics work can contribute to the elucidation of disease mechanisms and can help to discover and support the application of effective production systems. All these relate to animal welfare and to the production of safe and quality food from these animals.

As part of our group’s long-standing and continuing work in the field of small ruminant health management, we have performed a detailed evaluation of published works in which proteomics methodological approaches and technologies have been used to advance knowledge about small ruminants. The specific objective of this assessment was the presentation of quantitative characteristics on the content and the bibliometric details of publications on the use of proteomics methodological approaches and technologies in small ruminant work, while also highlighting their significance for small ruminant health and production.

The novelty of the present work refers to the systematic and detailed quantitative and qualitative evaluation of the application of proteomics methodologies in small ruminant research and clinical work. Emphasis is given to quantitative trends and bibliometric patterns of published studies. The study provides quantitative insights into research trends, emerging topics, influential researchers, and institutions, and, moreover, reveals research networks for potential collaborations. The present approach highlights the current state of the field, identifies gaps where proteomic tools remain underutilized, and indicates opportunities where proteomics work can contribute to innovation in veterinary science and safe food production.

Scientometrics can synthesize scientific literature within a discipline or a defined topic, with the aim of identifying trends and hotspots. The results of scientometric studies can facilitate a comprehensive understanding of the research landscape in the discipline or topic under study. The findings of such an analysis can show research themes, established and emerging research, as well as possible gaps in the relevant literature. The overview of scientometric studies can provide researchers with reference material and suggestions for possible collaborations, as well as ideas for future studies. In the fields of veterinary and animal science, scientometric studies have increased since 2020 [8].

## 2. Materials and Methods

### 2.1. Search Procedure

The Web of Science platform (www.webofknowledge.com; Clarivate Analytics, London, UK) was used for the search of relevant publications. In a previous study, we found that studies that employed the Web of Science database for data retrieval obtained significantly more records than studies that employed other databases [8].

A search using the following string was performed: TOPIC = [sheep OR ovine OR *Ovis aries* OR goat* OR caprine OR *Capra hircus*] AND TOPIC = [proteom*] (the asterisk served as a truncation symbol to include variations in the respective terms). The Web of Science Core Collection was used for this search, which expanded multiple disciplines through the inclusion of the Science Citation Index Expanded, the Emerging Sources Citation Index, the Social Sciences Citation Index, the Arts and Humanities Citation Index, the Conference Proceedings Citation Index, and the Book Citation Index. The search was performed on 18 March 2025 (‘freeze date’) and was repeated on 15 May 2025 to confirm that no additional records (i.e., delayed entries) had been added thereafter. Only records published up to 31 December 2024 were included in this study.

Thereafter, an initial document-type analysis of the records obtained was performed, during which only the following types of documents were included: ‘article’ and ‘review article’. Thus, the following categories of documents were excluded: (i) ‘meeting abstracts’, (ii) ‘corrections’, (iii) ‘editorial material’, and (iv) ‘book chapters’; the exclusion was performed by the Web of Science platform. Thus, 970 papers were retained for further screening and assessment individually (Table S1). The Web of Science tool for analysis of the language of these papers indicated that all these 970 papers (100.0%) were in the English language.

### 2.2. Paper Evaluation

Evaluation of the characteristics of the papers was performed by four people. Initially, the 970 papers were assessed by two people, who worked separately and independently to confirm that the papers included work related to sheep or goats. Papers that indeed included work related to sheep or goats (*n* = 543) were thereafter assessed by another two people, who also worked independently to confirm that the papers included work related to proteomics (*n* = 481) (Table S1). In case of disagreement between the two people who worked on each set of papers, the papers in which a disagreement was noted were evaluated by a senior author (author G.C.F. during the first screening: sheep or goat work, and author A.I.K. during the second screening: proteomics work). In such cases, the results returned by two of the three authors who evaluated each paper were taken into account. This occurred with two papers during the first screening (0.2%; kappa coefficient for inter-rater agreement: 0.998 (standard error: 0.001)) and with five papers during the second screening (0.9%%; kappa coefficient for inter-rater agreement: 0.99 (standard error: 0.004)).

Thus, after the above, 481 published papers remained and were included in the final evaluation [9] (Table S1). These 481 published papers were assessed individually. In each of these, the following details were recorded.

1. Type of published paper: original article or review.
2. Animal species referred to in the paper: sheep, goats, or both species.
3. Year of publication of paper and country—scientific establishment of origin of the paper (as indicated in the affiliation(s) of the author(s)).
4. For original articles only: (i) the type of work ((a) experimental work with animals, (b) field work, (c) computational work, (d) in vitro work), (ii) the topic of study ((a) sheep/goat production, (b) sheep/goat reproduction, (c) physiology, (d) animal diseases, (e) study that involved small ruminants as models for the study of various conditions in humans), (iii) the tissue analyzed by proteomics examinations, (iv) the methodological approaches for proteomics analyses employed, (v) the additional use of quantification analysis of the proteomics findings and (vi) the application of additional -omics technologies.
5. For original articles on sheep/goat production, the specific field (i.e., milk, meat, wool, hair, or horn production); for original articles on animal diseases, the specific disease studied; for original articles referring to small ruminants as models, the specific conditions studied.
6. Keywords listed in the published papers, journals in which the papers were published, authors of the papers (names and total numbers), references cited therein, and total number of citations received until the end of 2024 [8]. The number of citations received by the papers was normalized by calculating the average citations received annually per paper since the year of publication of each paper.

### 2.3. Data Management and Analysis

All data were systematically recorded and organized using Microsoft Excel (2019) (Microsoft Corporation, Redmond, WA, USA). Descriptive analysis was performed initially. The frequency of the various outcomes was evaluated in tables of cross-categorized frequency data using the Pearson chi-square test or the Fisher exact test, as appropriate. Comparisons between continuous data were performed using the Mann–Whitney test or the Kruskal–Wallis test. Linear regression analysis was used to establish associations with the year of publication of each paper, with year of publication considered as continuous. Spearman’s rank correlation analysis was performed as indicated, and the significance of the result was evaluated according to the critical values for *r*.

The outcomes ‘citations received by an original article yearly’ and ‘citations received by a review yearly’ were evaluated. Initially, univariable analysis was performed to assess potential associations with relevant parameters. Then, a multivariable model was developed for the above outcome, and parameters found with *p* < 0.20 in the univariable analyses previously carried out were included in this model. Progressively, parameters were removed from the model by using backwards elimination. The likelihood ratio test was performed to assess the *p* value of each parameter; among those found with *p* > 0.20, the one with the largest *p* was removed from the model. The procedure was repeated until no variable with *p* > 0.20 could be removed from the model. The parameters included in the final multivariable assessment are in Table S2. Subsequently, associations of the number of citations received by an original article and the parameters into that final multivariable assessment were evaluated by principal component analysis.

Statistical significance was defined at *p* < 0.05.

## 3. Results and Discussion

A total of 481 published papers included work on sheep and goats with proteomics methodologies. Most of these published papers were original articles (*n* = 448, 93.1%), and fewer were reviews (*n* = 33, 6.9%). Of these, most involved work on sheep (*n* = 253, 52.6%) and fewer on goats (*n* = 182, 37.8%) or on both animal species (*n* = 46, 9.6%).

The collection of information from published papers was performed by using the Web of Science platform. In a recent comparative study, we reported that scientometrics studies in the broad fields of veterinary or animal sciences, which employed the Web of Science for data extraction, retrieved a higher number of records (i.e., published papers) than studies that employed other similar platforms [8]. Moreover, this platform is publisher-neutral and unaffiliated to any journal publisher, with relevant decisions made by in-house editorial staff; this minimizes possible bias and conflicts of interest.

### 3.1. Year of Publication

#### 3.1.1. Findings

**Figure 1:**
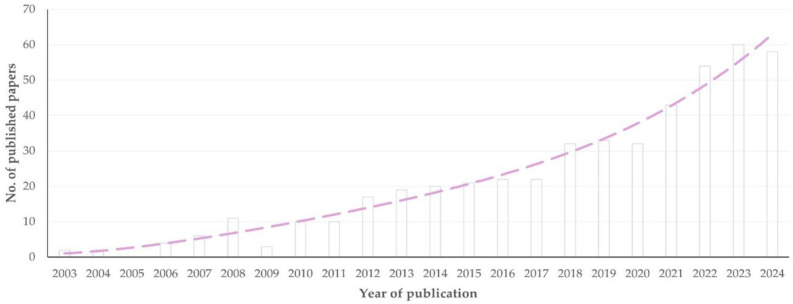
Bar plot of the number of published papers with sheep or goat work and proteomics in accordance with year of publication (dashed line is trendline).

**Figure 2:**
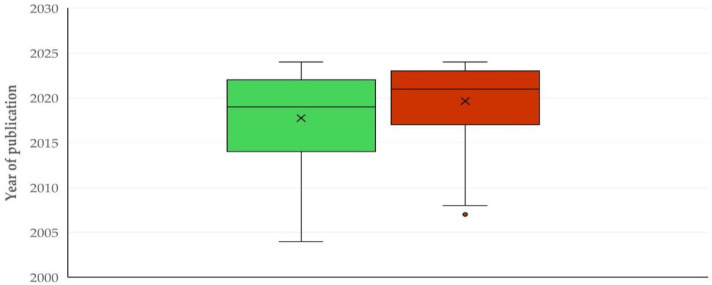
Box-and-whisker plot of year of publication of original articles with sheep (green; *n* = 272) or goat (brown; (*n* = 210)) work and proteomics.

The first paper on the use of proteomics methodologies in sheep or goat work was published in 2003. Subsequently, there was a clear and significant progressive increase in published papers with time (slope: 2.698 ± 0.206; *p* < 0.0001) (Figure 1). The median year of publication of reviews was older than that of original articles: 2017 (interquartile range (IQR): 8 years) versus 2020 (IQR: 6 years) (*p* = 0.035) (Figure S1). Furthermore, the median year of publication of original articles with goat work was more recent than that of the original articles with sheep work: 2021 (IQR: 6 years) versus 2019 (IQR: 8 years), respectively, (*p* < 0.0001) (Figure 2); however, there was no significant difference between the two species in the annual increase in publication of relevant articles (1.326 ± 0.102 versus 1.538 ± 0.174; *p* = 0.29) (Figure S2).

#### 3.1.2. Comments

The development of proteomics methodologies during the last 15 years has been reflected in the increasing number of relevant published papers (Figure 1). The expansion of the application of proteomics methodologies and of their incorporation into research protocols is also reflected in the increasing number of countries, from which relevant papers have originated on a yearly basis (Section 3.2.1.).

This is also seen in the publication of more recent papers with regard to goat than sheep work (Figure 2), which further confirms the dissemination of the usage of these technologies in additional animal species. As the goat industry has expanded and has become of more intensified management [10], consequently to increased demand for goat meat (due to its lean protein [11]) and milk (for use in cheese production and in infant-formula products [12]), the scientific community followed, responding to the higher necessity for study of the goat industry and its products.

### 3.2. Countries and Scientific Establishments of Origin

#### 3.2.1. Findings

The published papers originated from a total of 56 countries; the most frequent countries of origin of the papers were China (*n* = 211) and the United States of America (*n* = 50). Authors from 40 countries were first authors in the papers; these countries were mostly China (*n* = 206) and Italy (*n* = 39) (Table 1 and Table S3). The median number of countries of origin per published paper was 1 (IQR: 1, max.: 6). The most frequent combinations of countries in the authorship of published papers were as follows: United States of America and China (*n* = 13 papers) and United States of America and India (*n* = 4).

**Figure 3:**
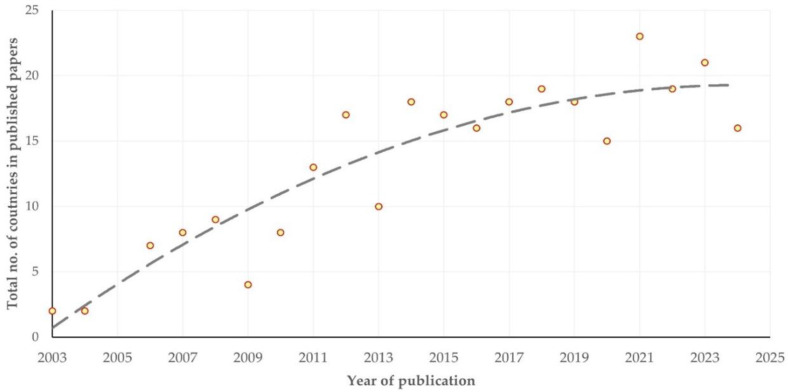
Scatter plot of the number of countries annually with published papers with sheep or goat work and proteomics (dashed line is trendline).

There was a clear progressive increase in the number of countries from which published papers originated (slope: 0.870 ± 0.109; *p* < 0.0001) (Figure 3). Among countries with the most published papers, there was a significant difference in the median year of publication of the papers in accordance with the country of affiliation of the first author (*p* < 0.0001). The earlier published papers originated from the United Kingdom (median year of publication: 2011 (IQR: 8 years)), whilst the most recently published ones originated from China (2022 (IQR: 4 years)) (Table S4).

Among the countries with the most published papers, there were differences in the animal species referred to in the respective papers: papers from Australia, France, New Zealand, Portugal, Spain and the United Kingdom included mostly sheep work (*p* < 0.045 for all comparisons), whilst articles from China and India included mostly goat work (*p* < 0.026 for both comparisons); papers from Brazil, Germany, Greece, Italy, and the United States were balanced between sheep or goat work therein (*p* > 0.13 for all comparisons) (Table 1).

The published papers originated from a total of 472 scientific establishments. Most of these establishments were universities (*n* = 322, 68.2%). The median number of scientific establishments per paper was 2 (IQR: 2). The scientific establishments with the most published papers are in Table 2. In total, 24 countries had at least one scientific establishment with over three published papers (Table S5); the 13 countries with the most (>10) published papers were all among these. There was a clear correlation between the number of scientific establishments (with ≥3 published papers) per country and the number of published papers from that country (*r_sp_* = 0.928, *p* < 0.0001) (Figure S3).

#### 3.2.2. Comments

It is evident that most published papers have originated from countries with large numbers of sheep and goats (Table 1). China is the country with the largest numbers of small ruminants internationally [13], and this is reflected in the highest number of published papers found in this study with origin from that country. The difference in the papers with origin from India (8 papers with sheep work, 3.2% of all papers, and 15 papers with goat work, 8.2% of all papers; Table 1) reflects the higher number of the latter species of animals in that country: 150 M goats (13.6% of total animals globally) versus 75.3 M sheep (5.7% of total animals globally) [13]. The four para-Mediterranean countries (France, Greece, Italy, Spain), which have a strong small ruminant industry with a primary orientation in dairy production, have also published a large proportion (25.4%) of the total papers found in this study. Another group of countries (Australia, New Zealand, United Kingdom), these with a significant sheep industry, have also contributed a significant proportion (18.5%) of the published papers.

The countries with a high number of published papers also had a correspondingly large number of scientific establishments (Figure S3). This can be explained because, for proteomics work, core facilities are necessary to perform the analytical process. Therefore, a large number of establishments equipped with appropriate infrastructure is necessary for a large research output from a country, as reflected in the present findings.

### 3.3. Type of Work and Topic of Study in Original Articles

#### 3.3.1. Findings

##### Type of Work

**Figure 4:**
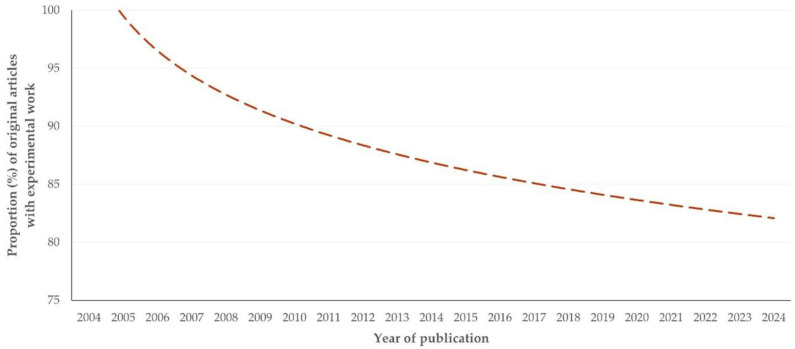
Scatter plot of the proportion of original articles presenting animal experimental work (*n* = 384) with sheep or goat work and proteomics, in accord with year of publication (dashed line is trendline).

Original articles included most frequently experimental work with animals (*n* = 384, 85.7%). Less often, they included field (*n* = 54, 12.1%), computational (*n* = 7, 1.6%), or in vitro (*n* = 3, 0.7%) work. There was no difference in the type of work, in accord with the animal species involved (*p* = 0.57). With time, there was a notable progressive reduction in the proportion of original articles presenting experimental work (slope: −0.867 ± 0.325, *p* = 0.011) (Figure 4).

There were also differences between countries in the type of work presented in original articles with respective origin (*p* < 0.0001 between countries). Although experimental work was most frequently included in original articles from all countries, the proportion of relevant articles was highest among papers from the United States (95.7%) and Australia (90.9%); the proportion of original articles with field work was highest in papers from Italy (28.9%) and New Zealand (25.0%); finally, the proportion of original articles with computational or in vitro work was highest in original articles from Greece (22.2% and 11.1%, respectively) (Table S6). The proportion of articles with experimental work from countries in the European Union was significantly lower than that of articles from all other countries of the world: 77.7% versus 88.7%, respectively (*p* = 0.003) (Figure S4).

##### Topics of Studies

**Figure 5:**
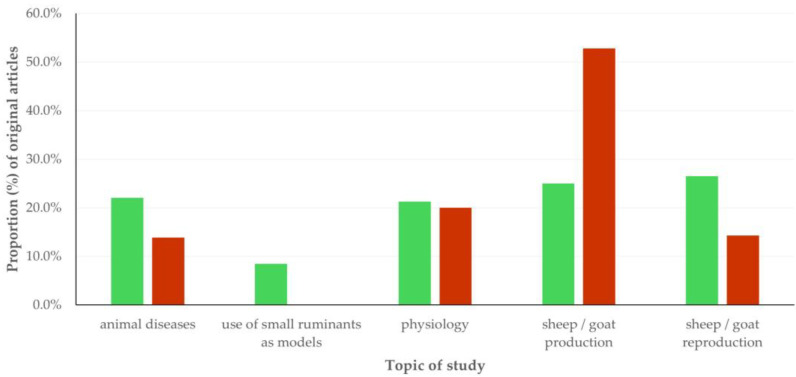
Bar plot of the proportions of original articles with sheep (green; *n* = 272) or goat (brown; *n* = 210) work and proteomics, in accordance with the topic of the study therein.

The topic of studies described in original articles most often was in the general field of sheep/goat production (*n* = 167, 37.2%). Less frequently, the topic of the studies was in the general field of sheep/goat reproduction (*n* = 98 (21.8%) original articles), in the general field of physiology (*n* = 93 (19.0%) original articles), and in the general field of animal diseases (*n* = 81 (18.0%) original articles). Finally, in 23 (5.1%) original articles, the topic of study involved small ruminants as models for the study of various conditions in humans. There were clear differences in the general field studied in the original articles in accord with the animal species referred to (*p* < 0.0001) (Figure 5, Table S7). Moreover, there was an association of the type of work with the topic of the study in original articles: 44.4% of articles with field work and 35.9% of articles with experimental work were included in the general field of sheep/goat production (*p* < 0.0001 for both comparisons). In contrast, for articles with computational or in vitro work, no such associations were found (*p* > 0.18 for both comparisons) (Table S8).

Among the original articles in the general field of sheep/goat production, most dealt with milk production (*n* = 92, 54.8%). Less often, original articles dealt with meat (*n* = 43, 25.6%), fiber (*n* = 19, 11.3%), wool (*n* = 16, 9.5%), or horn (*n* = 2, 1.2%) production.

In total, 27 different diseases were studied in the respective original articles (i.e., those in the general field of animal diseases) (Table S9). The individual disease studied most often was mastitis (*n* = 23, 28.4% of relevant articles); collectively, 24 (29.6%) original articles reported studies on various parasitic problems (coccidiosis, fasciolosis, haemonchosis, larval metacestodoses, teladorsagiosis, trichostrongylosis, and trypanosomiasis).

Moreover, a total of 22 human conditions were studied by using sheep or goats as models for their investigation (Table S10).

There were differences between countries in the topic of study presented in the original articles. Original articles from Italy were mostly in the general field of sheep/goat production (46.7%), original articles from Brazil mostly in the general field of sheep/goat reproduction (57.7%), original articles from Germany mostly in the general field of physiology (54.5%), original articles from Greece mostly in the general field of animal diseases (66.7%) and original articles from New Zealand mostly involved small ruminants as models for the study of various conditions in humans (25.0%) (*p* < 0.0001 between countries) (Table S11). Notably, there was a higher proportion of original articles in the general field of animal diseases with affiliation of a veterinary educational establishment (61.7%) than in all other topics of study (29.2%) (*p* < 0.0001) (Figure S5).

Articles from China predominated in all particular aspects of sheep/goat production studied: 100.0% of original articles on hair and horn production, 81.3% of original articles on wool production, 51.1% of original articles on meat production and 49.5% of original articles on milk production.

With regard to the various diseases studied, original articles from France, Greece, Italy, and Spain collectively accounted for 70.8% of articles on mammary infections (contagious agalactia, mastitis). In contrast, no clear pattern emerged regarding the country of origin of articles on parasitic infections.

##### Thematic Priorities Between Sheep and Goat Work

**Figure 6:**
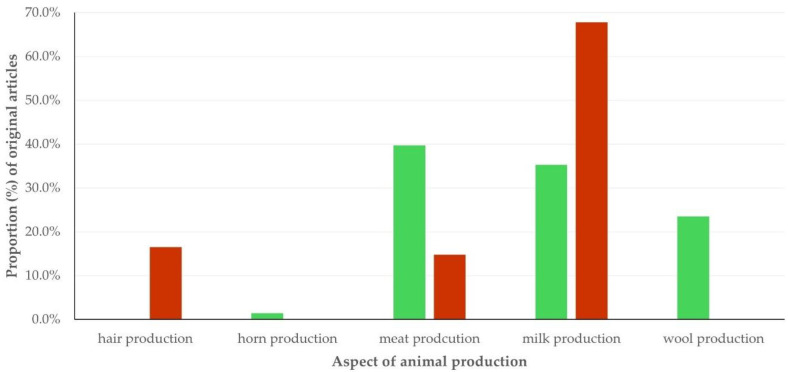
Bar plot of the proportions of original articles within the general field of animal production with sheep (green; *n* = 68) or goat (brown; *n* = 114) work and proteomics, in accord with the particular aspect of animal production studied therein.

There were clear differences in the particular aspect within the general field of sheep/goat production studied in the original articles, in accord with the animal species referred to; in sheep, most frequently it was meat production, in goats, most frequently it was milk production (*p* < 0.0001) (Figure 6, Table S12). There was a difference between articles referring to each of the two species in the diseases studied; articles with sheep work reported work on parasitic infections more often than papers with proteomics work on goats: 34.4% versus 10.0% (*p* = 0.013). All the 23 articles that reported studies of using small ruminants as models for human conditions referred to work performed in sheep only: 8.5% versus 0.0% (*p* < 0.0001). Finally, there was no difference between original articles in the reproduction, for focusing on specific aspects of the field, e.g., for studying rams/ewes or bucks/does more frequently (*p* = 0.34).

#### 3.3.2. Comments

The majority of the original articles presented experimental studies in various topics and fields related to sheep and goat work (Table S6). This is reasonable, because proteomics applications are high throughput technologies that can provide a large amount of information [14,15]. They can be used more efficiently in experimental settings, due to the highly dynamic and complex nature of the various proteomes, as these require precise experimental design for accurate ‘snapshots’ of the biological processes under investigation [14,15]. Indeed, proteomics methodologies fit in appropriately into experimental studies, as they focus to proteins within a biological system. Experimental work using proteomics allows the capture of real-time cellular activity, in controlled circumstances, at the same time studying complex interactions beyond the genome; this can be achieved, for example, by linking molecular data to processes in healthy and diseased animals [14,16].

Nevertheless, the findings also revealed that the proportion of experimental studies has progressively decreased (Figure 4). This may be considered as a consequence of the global trend for the decreasing animal experimentations and their substitution with alternative methods, which has developed in the light of efforts to improve welfare of animals used in experimental studies [17,18]. The significantly lower proportion of experimental studies with origin from countries of the European Union (Figure S4) can be explained by the comprehensive relevant legislation regarding the welfare of experimental animals enforced in those countries [19].

Most original articles presented work on the general field of sheep/goat production. Animal production refers to the general process and methodologies for keeping and breeding livestock for food production. The large extent and breadth of this field, which includes many sub-fields, e.g., animal nutrition, breed characterization, genetic improvement, quality of products from sheep and goats [20], explains the large number of relevant articles. Moreover, work on animal diseases requires particular infrastructure, for example, biosecurity-related installations, as well as veterinary monitoring of animals in the study, which explains the high proportion of articles on that field with an affiliation of a veterinary educational establishment (Figure S5).

The frequent study of mammary infections (mastitis and contagious agalactia) in articles that originated from the four para-Mediterranean countries (France, Greece, Italy, Spain), reflects the significance of dairy production in sheep and goats in these countries. Notably, Greece is the only country in Europe where milk production from small ruminants exceeds that from cows [21].

Parasitic infections are detrimental to small ruminant production and reproductive efficiency [22,23,24,25,26]. Hence, there is increased interest in their study, in order to implement new preventive strategies for their control.

In Greece, mastitis and cerebral coenurosis were reported by farmers as the most important and the second most important health problems in adult animals and in replacement animals, respectively [27]. Moreover, in a multinational survey performed in France, Greece, Italy, and Spain, mastitis was also declared by farmers and professionals as the most important health issue in sheep flocks [28]. In Australia, New Zealand and the United Kingdom, small ruminant farmers considered that nematode parasitism was the most significant animal health problem in their farms [29,30,31]. These studies have underlined the significance of health problems in sheep and goat farms; their results are similar to ours with the current findings regarding the study of diseases by means of proteomics technologies in sheep and goat farms (Table S10).

### 3.4. Tissues Analyzed

#### 3.4.1. Findings

In total, 51 different tissues were analyzed in the proteomics studies evaluated (Table S13). The tissues used most frequently in the respective studies, were milk (in 80 original articles, 17.9%), blood (in 46 articles, 10.3%), muscle (in 41 articles, 9.2%), milk fat globule membrane (in 29 articles, 6.5%), semen (in 24 articles, 5.4%), skin (in 22 articles, 4.9%), ovary (in 21 articles, 4.7%), and liver (in 20 original articles, 4.5%).

In studies on milk production, milk was the tissue analyzed most frequently (in 67.8% of relevant original articles); other tissues analyzed were milk fat globule membrane (in 26.7% of relevant original articles), colostrum (in 8.9%), mammary tissue (in 7.8%), and blood or liver (in 1.1% of relevant original articles). In studies on meat production, muscle was the tissue analyzed most frequently (in 83.7% of relevant original articles); other tissues analyzed were adipose tissue (in 9.4% of relevant original articles) and bone, embryo, heart, or liver (in 2.4% of relevant original articles).

In studies on mastitis, milk was also the tissue analyzed most frequently (in 60.9% of relevant original articles); other tissues analyzed were milk fat globule membrane (in 17.4%), blood (in 13.0%), and mammary tissue (in 8.7% of relevant original articles). In studies on parasitic infections, blood was the tissue analyzed most frequently (in 37.5% of relevant original articles); other tissues used were abomasum and liver (in 20.8%), lung (in 8.3%) and bile, bile duct, brain, intestine, lymph-node or peritoneal fluid (in 4.2% of relevant original articles).

There were differences in the tissues analyzed between studies with sheep or goat work. First, in sheep work 51 different tissues were analyzed, whilst in goat work 33 different tissues were analyzed (*p* < 0.0002). Second, there were also differences in the type of tissue analyzed (*p* < 0.0001): in sheep work, blood (*n* = 34), milk (*n* = 29) or muscle (*n* = 24) were analyzed most frequently, whilst in goat work milk (*n* = 63), milk fat globule membrane (*n* = 23) or muscle (*n* = 18) were analyzed most frequently (Table S14).

#### 3.4.2. Comments

The variety of tissues analyzed in the studies evaluated reflects the number of topics and subtopics on which relevant work has been performed. In general, the most frequently used tissues can be included in four large groups. The first group would include milk and tissues of mammary origin, which were used mostly in studies on milk production; the second group would include meat and adipose tissue, which were used mostly in studies on meat production; the third group would include tissues of genital system (ovary, semen, etc.), which were used in studies on the general field of sheep/goat reproduction; the fourth group would include blood, which was used in a variety of studies, e.g., studies on the general field of physiology, studies on parasitic infections, etc. In total, in 67.4% of original articles, tissues within the above four groups, were studied.

The choice of these tissues in proteomics analyses reflects the type of work performed and the research objectives of the scientists. Moreover, some of these tissues could be obtained with no invasive techniques, e.g., milk and milk-related tissues, semen, or with minimal disturbance to animals, e.g., blood, which makes these tissues easy for collection and handling, at the same time maintaining the relevant animal welfare regulations, as discussed above.

### 3.5. Proteomics Methodological Approaches and Technologies Used

#### 3.5.1. Findings

In total, 11 different proteomics methodological approaches and technologies were used in the studies reported in the original articles. Liquid chromatography–tandem mass spectrometry (LC-MS/MS) was the technology employed most often, specifically in work described in 237 (52.9%) original articles (Table 3). There was no difference in the use of proteomics methodological approaches and technologies in accord with animal species (sheep or goats) involved in the study (*p* = 0.19), but there was a significant difference in the median year of publication of papers in accord with the proteomics methodological approach and technology employed (*p* < 0.0001) (Table 3, Figure S6). There was also a significant association of the proteomics methodological approaches and technologies used with the type of work carried out (*p* < 0.0001) (Table S15), as well as with the topic of study (*p* = 0.019) (Table S16).

**Figure 7:**
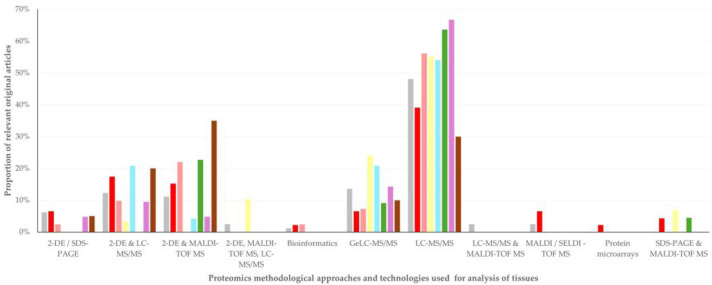
Bar plot of the proportions of original articles with sheep or goat work and proteomics, in accord with the proteomics methodological approaches and technologies ^1^ used in the respective studies for the analysis of milk (gray; *n* = 81), blood (red; *n* = 46), muscle (pink; *n* = 41), milk fat globule membrane (yellow; *n* = 29), semen (light blue; *n* = 24), skin (green; *n* = 22), ovary (purple; *n* = 21), and liver (brown; *n* = 20) (i.e., the eight most frequently analyzed tissues ^2^). ^1^ 2-DE: two-dimensional gel electrophoresis, SDS-PAGE: Sodium dodecyl–sulfate polyacrylamide gel electrophoresis, LC-MS/MS: liquid chromatography–tandem mass spectrometry, MALDI-TOF MS: matrix-assisted laser desorption/ionization coupled to time-of-flight mass spectrometry, GeLC-MS/MS: polyacrylamide gel electrophoresis followed by liquid chromatography-tandem mass spectrometry SELDI-TOF MS: Surface-enhanced laser desorption/ionization time-of-flight mass spectrometry. ^2^ Tissues analyzed in studies reported in ≥20 original articles.

There were significant differences in the use of the various methodological approaches and technologies, in accord with the tissue analyzed in the study (*p* < 0.0001) (Figure 7, Table S17). LC-MS/MS on its own was employed for the analysis of 26 of the 28 tissues (92.9%) evaluated in studies in over three original articles. LC-MS/MS was most frequently employed in the analysis of brain, embryo, fibers, heart and spleen (in ≥75.0% of respective studies), but not in the analysis of cerebrospinal fluid and vaginal excretions (in 0.0% of respective studies). These two tissues were analyzed mostly with 2-DE and LC-MS/MS (50.0% of analyses of cerebrospinal fluid) or with GeLC-MS/MS (50.0% of analyses of vaginal excretions). The median year of publication of the original articles with studies of analysis of these two tissues (i.e., cerebrospinal fluid or vaginal excretions) was 2012.5 (IQR: 8 years), whilst the median year of publication of all other original articles was 2020 (IQR: 6 years) (*p* = 0.013).

#### 3.5.2. Comments

The current findings indicate a clear shift in the methodologies followed in proteomics work to advanced mass spectrometry (MS) platforms (Table 3, Figure S6), with LC-MS/MS being the dominant workflow across tissues and topics (Figure 7, Table S17). This reflects the global proteomics trends, in which MS-based strategies have replaced gel-based techniques and approaches and have established shotgun LC-MS/MS as the most common approach for deep profiling [32,33,34]. Indeed, very few matrices (e.g., cerebrospinal fluid, vaginal excretions) still relied on hybrid gel/MS methodological approaches, whilst most recent works used LC-MS/MS on its own.

With respect to the proteomics analysis of cerebrospinal fluid and vaginal excretions, it is noted that gel-based proteomic approaches (e.g., 2-DE) have been widely applied in biofluids with low protein content and high dynamic range, as these two tissues, where pre-fractionation enriched low-abundance proteins and facilitated biomarker discovery. Although currently, proteomics work on these two tissues relies primarily on LC-MS/MS–based workflows, gel-based methods can usefully serve as complementary approaches for the analysis of isoforms and post-translational modifications [35,36]. The above are confirmed by the significantly older publication years of the original articles with analysis of cerebrospinal and vaginal excretions.

### 3.6. Quantification Analysis of Proteomics Findings

#### 3.6.1. Findings

Quantification analysis of proteomics findings was reported in 190 original articles (42.4%). The technique employed most frequently for quantification analysis was isobaric tagging methods (*n* = 96); other techniques employed less often included Western blot (*n* = 58), label-free proteomics (*n* = 40), and targeted mass spectrometry quantification (*n* = 27).

**Figure 8:**
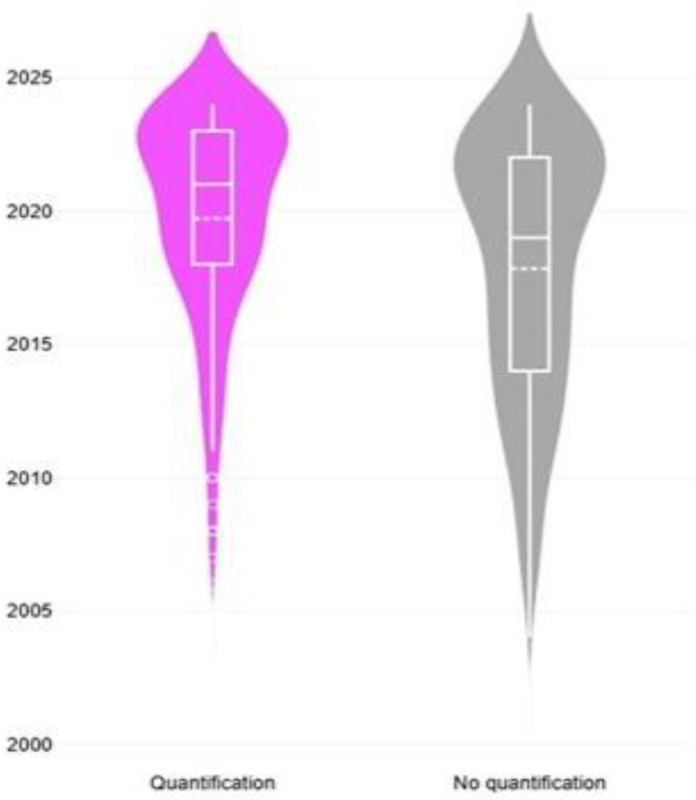
Violin plot of year of publication of original articles with sheep or goat work and proteomics, in accord with the inclusion (purple; *n* = 190) or not (gray; *n* = 258) of quantification analysis.

**Figure 9:**
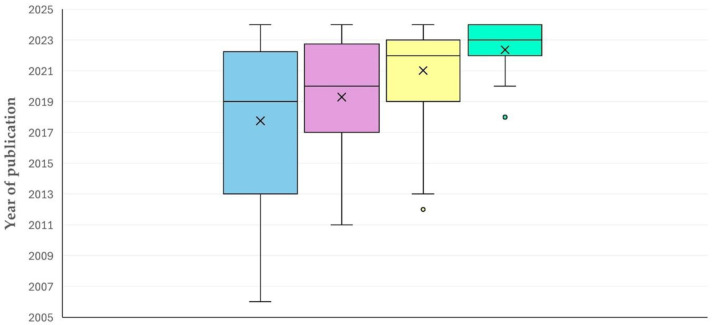
Box-and-whisker plot of the year of publication of original articles with sheep or goat work and proteomics, in accord with the technique employed for quantification analysis ^1^. ^1^ blue: Western blot (*n* = 58), purple: label-free proteomics (*n* = 40), yellow: isobaric tagging methods (*n* = 96), light green: targeted mass spectrometry quantification (*n* = 27).

The median year of publication of original articles that reported quantification analysis, was more recent than that of original articles that did not report such an approach: 2021 (IQR: 5 years) versus 2019 (IQR: 8 years) (Figure 8). Moreover, there was a significant difference in the median value of the year of publication of the articles in accord with the specific quantification analysis employed; for Western blot it was 2019 (IQR: 4 years), for label-free proteomics 2020 (IQR: 5 years), for isobaric tagging methods 2022 (IQR: 9 years) and for targeted mass spectrometry quantification it was 2023 (IQR: 1.5 years) (*p* < 0.0001) (Figure 9).

Furthermore, there was also a significant association of the proteomics methodological approaches and technologies employed in a study with performing the same study quantification analysis (*p* < 0.0001). For example, the application of LC-MS/MS was the one most associated with quantification assessment (57.2% of cases).

#### 3.6.2. Comments

Alongside this technological transition, quantitative proteomics work has also expanded. The popularity of these methods lies in their ability to compare multiple samples or conditions simultaneously, making them particularly useful for studies tracking protein expression across tissues, time points, or health status. In particular, multiplexed isobaric labeling (TMT/iTRAQ), which enables robust, multi-group comparisons and can support more complex experimental designs [37] was seen as the most popular relevant approach. This observation is in line with reports that isobaric labeling has become a key tool for relative protein quantification in the past decade [37]. The appeal of using these methods is their multiplexing capacity and workflow flexibility, as these enable robust comparison of protein abundance across treatment/experimental groups within a single MS run [37]. The prominence of isobaric tagging in recent sheep and goat studies (Figure 9) indicates that researchers have pursued complex experimental designs, for example, assessing protein expression changes across various tissues or time points in health and disease that can benefit from multiplexed quantitation. This can be another factor that contributed to the reduction in animal experimentation studies: as powerful analytical tools have become available for research work, scientists can obtain useful data from a variety of sources, thus reducing the need to perform animal experiments for obtaining clear and powerful results.

Targeted MS (SRM/PRM) has also emerged as a post-discovery, high-specificity solution for biomarker verification and precise protein quantification [38,39,40] with growing prospects for applications in disease monitoring, as well as in livestock breeding and nutrition [7].

### 3.7. Concurrent Use of Additional -Omics Technologies

#### 3.7.1. Findings

In 82 original articles (18.3%), the use of additional -omics technologies was reported. The -omics technology reported most often in conjunction with proteomics work, was transcriptomics (*n* = 55, 67.1% of relevant original articles); other methods reported less often included metabolomics (*n* = 25, 30.5%), genomics (*n* = 10, 12.2%) and metagenomics (*n* = 3.7%) and lipidomics and proteogenomics (*n* = 1 each, 1.2% of relevant original articles).

The median year of publication of original articles that reported use of additional -omics technologies was 2022 (IQR: 3 years) versus a median year of 2019 (IQR: 7 years) for the publication of original articles that did not report such a use. There was also a significant association of the proteomics methodological approaches and technologies employed with use in the same study of other -omics technologies (*p* < 0.0001). For example, the application of LC-MS/MS was the one mostly associated with quantification assessment (28.4% of cases).

#### 3.7.2. Comments

The rising integration of proteomics with other -omics technologies, in particular, transcriptomics and metabolomics, signals a shift toward systems biology frameworks that are able to capture better the multifactorial traits relevant to small ruminant health and production [41,42].

Thus, it is apparent that the proteomics field for small ruminant work is moving towards a systems biology paradigm. This shift towards multi-omics approaches reflects first the recognition that complex livestock traits require systems-level approaches and also the technological advances in bioinformatics.

### 3.8. Keywords

#### 3.8.1. Findings

The published papers included a total of 2211 keywords overall. The median value was 5 (IQR: 2) keywords per paper. These corresponded to 1130 unique keywords (Table S18). There were 17 keywords, which appeared in over 10 published papers (Table 4, Figure S7), among which ‘proteomics’, ‘sheep’ and ‘proteome’ were the ones listed more often.

**Figure 10:**
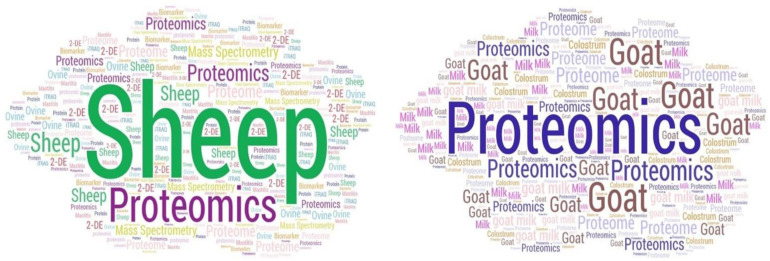
Word cloud of the most frequently occurring keywords in published papers with sheep (**left**) or goat (**right**) work and proteomics.

There were differences in the keywords featured in published papers involving sheep or goat work (Figure 10). Published papers involving sheep work featured most frequently the keywords ‘sheep’ (*n* = 79), ‘proteomics’ (*n* = 76) and ‘proteome’ (*n* = 29), while papers involving goat work featured most frequently the keywords ‘proteomics’ (*n* = 69), ‘goat’ (*n* = 39) and ‘proteome’ (n = 20). Moreover, there was a small, but statistically significant, positive correlation between the number of keywords listed per published paper and the year of publication of the respective paper (*r_sp_* = 0.095, *p* = 0.038) (Figure S8).

Among the keywords with ≥4 mentions in published papers (*n* = 98), most related to sheep or goats and their management (*n* = 57, 58.2%) with a total of 451 mentions (47.3%) (median value: 5). Fewer keywords related to proteomics work (*n* = 33, 33.7%) with a total of 430 (45.1%) mentions (median value: 6) or to both categories (*n* = 8, 8.1%) with a total of 73 mentions (7.6%) (median value: 9) (*p* < 0.0001 between proportions, *p* = 0.24 between median values).

#### 3.8.2. Comments

The number of keywords increased progressively with the year of publication, which may indicate the complexity of work performed in the various studies (Figure S8). Thus, researchers had to include more keywords to better describe the topic of their work.

A finding in support of the hypothesis presented above (Section 4.1) regarding the publication of more recent papers with regard to goat than sheep work is the difference in the predominant keywords between papers with sheep or with goat work. In the former, the word ‘sheep’ was the most frequent, whilst in the latter, the respective word was ‘proteomics’ (Figure 10). This may indicate that in sheep-related papers (which also have an older median publishing date), proteomics has not been the predominant feature, in contrast to the more recent goat-related publications, in which the use of proteomics technologies is considered more important than other topics in the published papers.

The increased number of keywords related to sheep or goats and their management, in comparison to keywords related to proteomics work may indicate that, overall, the main objective of authors in these papers was to showcase the applied facets of their work (e.g., production-related, specific diseases, etc.) rather than the methodological approaches.

### 3.9. Journals

#### 3.9.1. Findings

The papers were published in 198 different journals. The six journals, in which papers were published most frequently, were *Journal of Proteomics* (*n* = 40, 8.3%), *Animals* (*n* = 20, 4.1%), *International Journal of Molecular Sciences* (*n* = 16, 3.3%), *Journal of Dairy Science* (*n* = 15, 3.1%), and *Proteomics* and *Food Chemistry* (*n* = 14 in each, 2.9%) (Table S19).

Among the 198 journals, there were 12 (6.1% of journals) (*Analytical and Bioanalytical Chemistry*, *Analytical Biochemistry*, *Current Proteomics*, *Electrophoresis*, *Journal of Microbiological Methods*, *Journal of Proteome Research*, *Journal of Proteomics*, *Molecular and Cellular Proteomics*, *Proteome Science, Proteomics*, *Proteomics Clinical Applications*, and *Rapid Communications in Mass Spectrometry*) classified into the category ‘Biochemical Research Methods’ in the Web of Sciences. In that proportion (6.1%) of journals (*n* = 12), a significantly higher proportion (16.4%) of papers (*n* = 79) was published (*p* = 0.0003).

Most original articles were published in *Journal of Proteomics* (*n* = 38, 8.5%) and *Animals* (*n* = 18, 4.0%), whilst most review papers were published in *Parasite Immunology* (*n* = 3, 9.1%) and fewer in *Journal of Proteomics* and *Animals* (*n* = 2 each, 6.1%). When evaluating the type of published papers in journals with ≥4 published papers in total, there was no significant difference in the frequency of original articles or reviews published in each journal (*p* = 0.07). Also, there was no difference, in the proportion of original articles published in journals of the category ‘Biochemical Research Methods’ or in journals outside this category: 92.4% versus 93.3%, respectively (*p* = 0.78).

When considering the countries with the most published papers, *Journal of Proteomics* emerged with most published papers from seven of these 13 countries (Table 5). When considering the topic of the study described in the respective original articles, again *Journal of Proteomics* emerged with most published papers in four of these topics (Table 6).

**Figure 11:**
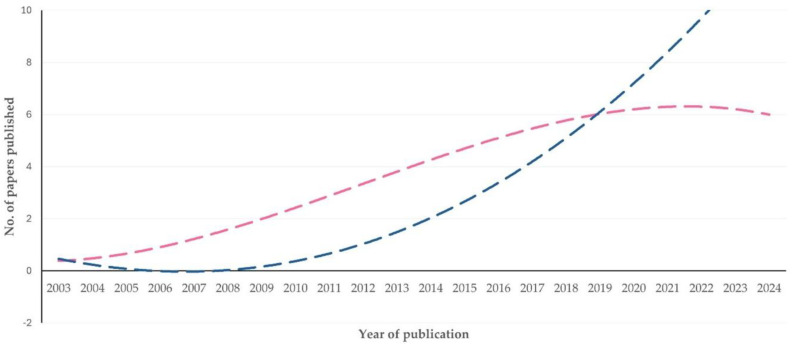
Scatter plot of the number of published papers with sheep or goat work and proteomics, in accord with year of publication in the five journals with most papers and content on proteomics–molecular studies ^1^ (pink line; *n* = 82) and in the eight journals with most papers and content on clinical–health management studies ^2^ (blue line; *n* = 77) (dashed lines are trendlines). ^1^ *Journal of Proteomics*, *International Journal of Molecular Sciences*, *Proteomics*, *Electrophoresis*, *Journal of Proteome Research*; ^2^ *Animals*, *Journal of Dairy Science*, *Frontiers in Veterinary Science*, *Small Ruminant Research*, *Journal of Dairy Research*, *Reproduction in Domestic Animals*, *Research in Veterinary Science*, and *Animal Reproduction Science*.

A comparison between (i) the number of papers published annually in the five journals with ≥5 papers and content on proteomics–molecular studies (*Journal of Proteomics* (*n* = 40), *International Journal of Molecular Sciences* (*n* = 16), *Proteomics* (*n* = 14), *Electrophoresis* (*n* = 6) and *Journal of Proteome Research* (*n* = 6), i.e., total number of papers *n* = 82) versus (ii) the number of papers published annually in the eight journals with ≥ five papers and content on clinical–health management studies (*Animals* (*n* = 20), *Journal of Dairy Science* (*n* = 15), *Frontiers in Veterinary Science* (*n* = 10), *Small Ruminant Research* (*n* = 9), *Journal of Dairy Research* (*n* = 6), *Reproduction in Domestic Animals* (*n* = 6), *Research in Veterinary Science* (*n* = 6), and *Animal Reproduction Science* (*n* = 5), i.e., total number of papers *n* = 77), indicated the emergence of a tendency for a progressive and gradual increase in the number of papers published in the latter cohort of journals (slope: 0.329 ± 0.056 versus 0.601 ± 0.083, respectively, for each of the two cohorts; *p* = 0.09) (Figure 11). Moreover, there was a clear difference in the median year of publication of papers in the two cohorts of journals: 2018 (IQR: 7 years) versus 2021 (IQR: 4 years) (*p* = 0.0001) (Figure S9).

#### 3.9.2. Comments

The findings show that, overwhelmingly, *Journal of Proteomics* has been the predominant medium for the dissemination of relevant results [43]. First, most papers were found to have been published in this journal; second, the journal has been used for publication of results in seven of 13 countries, with most papers published (Table 6) and in four of the five topics of study in the papers (Table 7). In a previous study that we performed on the use of proteomics in mastitis (which included primarily cattle-related papers), the *Journal of Proteomics* was also found to be the leading journal for publishing relevant results [44]. *Journal of Proteomics* is the official journal of the European Proteomics Association, which confirms its primary role in the field, as well as its ability to attract high-quality submissions. As such, the journal covers a wide range of topics within the broad field of proteomics work, from fundamental works in basic sciences to translational research and clinical studies [43]. These attributes can explain the popularity of the journal and its emergence as the primary medium for the dissemination of the results of research analyzed in the present study.

However, a shift in the scientific journals, where the relevant papers have been published, also emerged: from journals with content on proteomics–molecular studies to journals with content on clinical–health management studies (Figure 11), with the papers published in the latter cohort of journals having a more recent median year of publication (Figure S9).

One can hypothesize that earlier studies published in specialized journals focused on methodological developments and pioneering applications of the technologies; the more recent papers, published in clinically oriented journals, reflect the wider application of the already established technologies. This further indicates that with the expansion of the methodologies, their incorporation into scientific protocols has become more common and frequent.

Moreover, journals in the latter cohort have published Special Issues for papers with proteomics work. For example, in the recent past, *Animals* has published two Special Issues titled ‘Proteomics in veterinary research and zoonotic diseases’ and ‘Genetics, genomics, transcriptomics, proteomics, health and product quality of small ruminants’. Likely, these Special Issues acted as an attraction for submission of relevant manuscripts, that way increasing the number of relevant published papers in that journal [45].

### 3.10. Authors

#### 3.10.1. Findings

There were 3689 authors in total in the published papers, corresponding to 2451 distinct (unique) authors. There were also 481 first authors in total in the published papers, corresponding to 380 distinct authors. The median number of authors was 7 (IQR: 5) per published paper, with a minimum number of one author in four published papers and a maximum number of 21 authors in one published paper.

**Figure 12:**
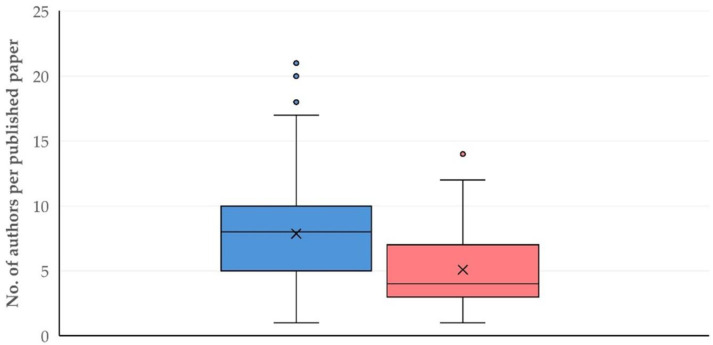
Box-and-whisker plot of the number of authors for original articles (blue; *n* = 448) or reviews (red; *n* = 33) with sheep or goat work and proteomics.

There was no significant difference in the number of authors per published paper in accord with the animal species dealt with in the published papers (sheep: 8 (IQR: 5), goats: seven (IQR: 4), *p* = 0.15), but there was a clear and significant difference between original articles and reviews in the number of authors: eight (IQR: 5) versus four (IQR: 4), respectively (*p* < 0.0001) (Figure 12). Moreover, there was a mild but significant, positive correlation of the number of authors per paper with the year of publication (slope: 0.096 ± 0.030, *p* = 0.003) (Figure S10).

There was a difference in the number of authors per published paper, in accord with the country of origin; papers from the United Kingdom had the smaller median value: 6 (IQR: 4), and papers from Brazil had the highest median value: 11 (IQR: 4) authors per published paper (*p* = 0.005 between countries) (Table S20). Further, there was also a difference in the number of authors per published paper, in accord with the topic of the study; papers with studies involving small ruminants as models for the study of various conditions in humans had the highest median value: 10 (IQR: 7) (*p* = 0.005 between the topics of study) (Table S21).

There were 44 people with authorship in over five published papers, of whom 6 were authors in over 10 published papers. There were also 13 people with over two published papers as first authors, of whom four also had over four papers published as first authors. Six authors among the ones with >2 published papers as first authors were also authors in >4 published papers; among these people, the proportion of papers published as first authors among all their papers varied from 40.0% to 100.0% (median value: 61%).

**Figure 13:**
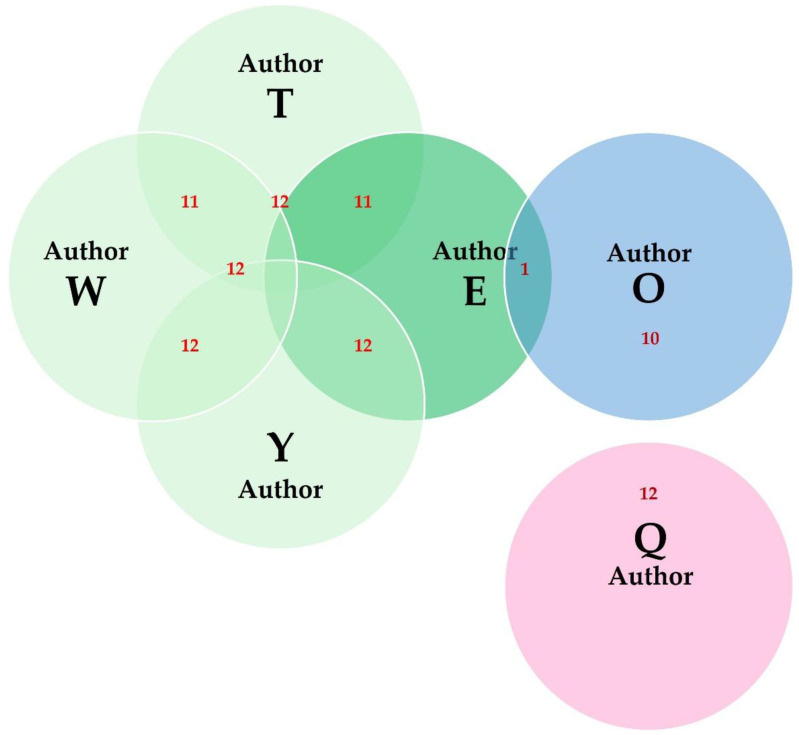
Venn diagram of joint authorships among the six authors with >10 published papers with sheep or goat work and proteomics (descriptors of authors not corresponding to their surnames).

The six authors with over 10 published papers cumulatively published a total of 37 papers. There were joint authorships in these papers, between five of these authors; these were extensive between four of these authors and occasional between two of them (Figure 13). Five of these authors were located in Europe and one in America. There were some common features in the papers published by these authors. The published papers presented work on sheep and goats and were mostly original articles; further, their published papers described mostly experimental work. However, there were also some differences: five of the authors carried out mainly work on animal diseases (specifically, on mammary proteomics) and the sixth author on sheep/goat reproduction (specifically, on genital system (ovary, semen) proteomics). Correspondingly, five of the authors used in the proteomics analyses mostly tissues related to the mammary gland (e.g., mammary tissue or milk), while one used tissues from the genital system (e.g., ovary or semen); further, five of the authors published most frequently in *Journal of Proteomics* and one in *Reproduction in Domestic Animals*; moreover, four of the authors used mostly LC-MS/MS, one used mostly 2-DE and MALDI-TOF MS and one used mostly GeLC-MS/MS in the proteomics analyses. Finally, the median year of publication of the respective papers was 2015.5 for four authors, 2020 for one, and 2020.5 for another author (*p* = 0.005).

#### 3.10.2. Comments

The highest number of authors per published paper was noted among those in which sheep or goats were used as models for the study of diseases of people (Table S21). This relates to the necessity for larger groups of researchers, with the participation of veterinarians, animal scientists, proteomics researchers (e.g., biomedical scientists or biochemists), as well as medical scientists, to cover the part of the study related to people. This is an example of translational research [46], which bridges the gap from early-stage findings of scientific work (e.g., under in vitro conditions) through work in animal models to actually producing clinical interventions for human benefit [47].

The progressive increase in the number of authors per published paper (Figure S10), first, is in line with the general tendency for increasing the number of authors in publications across all scientific disciplines [48]. Furthermore, it may also indicate that proteomics researchers have teamed up with scientists working in applied sciences in order to expand the use of proteomics methodologies in practical applications.

The results indicated a prolific group of authors based in Europe, with an occasional collaboration with another author, also based in Europe (Figure 13). These five authors have been active in mammary proteomics, working on the study of milk production and of mammary diseases in sheep and goats, which can explain their collaboration. The sixth author among those with a high number of published papers was based in America, which, reasonably, limited the possibilities for collaboration. All other authors had a smaller amount of published papers, which can reflect the high costs of setting up the necessary infrastructure (i.e., proteomics-related equipment) and carrying out that type of research [40,49], as well as the highly specialized knowledge required for successful performance of proteomics work [40].

### 3.11. References and Citations

#### 3.11.1. Findings

##### References

The median number of references per published paper was 50 (IQR: 30). Reviews had a higher median number of references than original articles: 127 (IQR: 47) versus 49 (IQR: 28), respectively (*p* < 0.0001). There was no association between the number of references and the animal species referred to in the published papers: 51 (IQR: 34) for sheep and 49 (IQR: 31) for goats (*p* = 0.41). There was also neither correlation between the number of cited references/the number of keywords per published paper (*r_sp_* = 0.007, *p* = 0.89), nor between the number of references/the year of publication of the papers (*r_sp_* = 0.078, *p* = 0.09).

**Figure 14:**
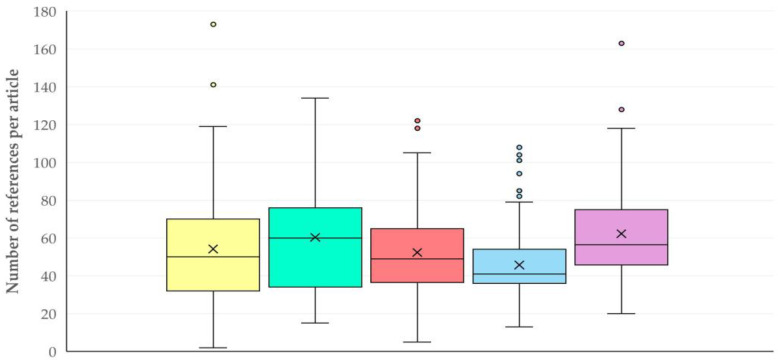
Box-and-whisker plot of the number of cited references per original article with sheep or goat work and proteomics, in accordance with the topic of study ^1^ described therein. ^1^ Yellow: animal diseases (*n* = 81), green: models for the study of various conditions in humans (*n* = 23), red: physiology (*n* = 93), blue: sheep/goat production (*n* = 167), purple: sheep/goat reproduction (*n* = 98).

For original articles, no significant difference was found in the number of references in accordance with the type of work performed (*p* = 0.59) (Table S22). However, differences were evident in accordance with the topic of the study: articles with studies that involved small ruminants as models for the study of various conditions in humans, had the highest median number of references: 60 (IQR: 33.5), whilst articles in the general field of sheep/goat production had the smallest: 41 (IQR: 17.5) (*p* < 0.0001) (Figure 14, Table S23).

##### Citations

The median number of citations per published paper was eight (IQR: 17, min: 0–max: 268). The *h*-index of this set of published papers was 42 (*m*-index: 2), and the *i_10_*-index was 212. The median number of annual citations per published paper was 2.0 (IQR: 3.0, min: 0.0–max: 18.9).

There was no significant difference between original articles and reviews in the median number of annual citations per published paper: 2.0 (2.9) versus 2.3 (4.9) (*p* = 0.25). The results of the univariable analyses for predictors associated with the number of annual citations per original article or review are in Table S24 and S25.

**Figure 15:**
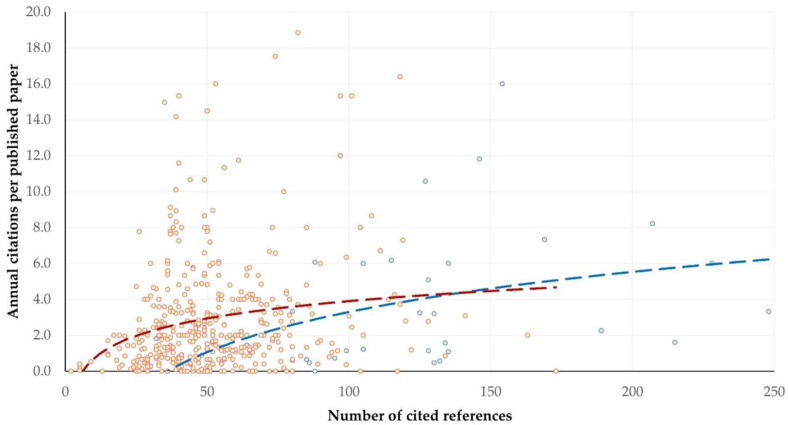
Scatter plot of the number of annual citations received by original articles (pink-yellow dots; *n* = 448) or reviews (black-light blue dots; *n* = 33) with sheep or goat work and proteomics, and the number of references cited in the respective papers (dashed lines are respective trendlines).

**Figure 16:**
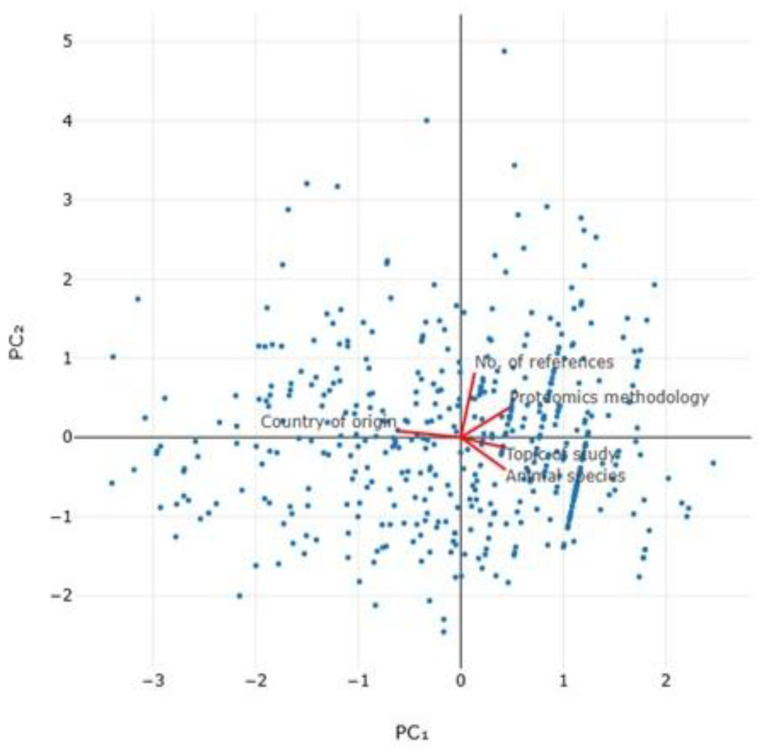
Bi plot of results of principal component analysis for the number of annual citations in original articles with sheep or goat work and proteomics (vectors from left to right: country of origin, no. of references, proteomics methodology, topic of study, animal species).

For original articles, three variables emerged in the multivariable analysis with significant association with the number of annual citations: (i) the number of references cited in the original article (*p* < 0.0001) (Figure 15), (ii) the topic of study described in the article (*p* = 0.015) and (iii) animal species involved in the study (*p* = 0.044) (Table 7). There was, nevertheless, a tendency for association also with the proteomics methodological approaches and technologies employed in the study described in the article (*p* = 0.06) and the country of origin of the original articles (*p* = 0.08). The principal component analysis for the number of annual citations in original articles indicated that the two principal components accounted for 49.4% of the variation (Figure 16 and Figure S11, Table 8). For reviews, the only variable that was found with significant correlation with the number of annual citations was the number of references cited in the paper (*r_sp_* = 0.462, *p* = 0.007) (Figure 15).

#### 3.11.2. Comments

The significant association of increased citations per published paper likely reflects an increased amount of information and ideas developed within published papers, for which appropriate referencing has also been carried out. Such papers contain more and more diverse data, which leads to the development of more ideas, which require a larger number of cited references for support [50,51,52]. Fox et al. [51] also reported that longer papers (for which we can postulate that they present more ideas therein) generally receive more citations, which can align with the larger number of cited references therein. Nevertheless, there is also the possibility that such papers become more visible in searches in citation databases, and thus cited more frequently, because of the longer reference lists [53].

## 4. Epimeter

The emergence and development of the various proteomics applications have created an explosion in high-throughput technologies. These were used initially in human studies and were later transferred to veterinary and animal science research. These studies included analysis of genomes primarily, as well as of proteomes and transcriptomes, by means of which opportunities for better insights into molecular pathways have arisen [54]. Their application of research and clinical work on sheep and goats has contributed to the health management of small ruminants.

### 4.1. Milk Production

In general, most original articles about milk production in the broad field of sheep/goat production present descriptive studies on the milk of ewes and does. These studies have shown detailed information about the proteome of milk in various breeds of sheep or goats [55,56,57,58]. The studies have included work and protein characterization on milk, as well as on colostrum [59,60,61,62]. Other studies have presented comparative protein work on the milk of various animal species, including sheep and goats [63,64,65]. The proteome of the milk fat-globule membrane has also been studied extensively across various sheep and goat breeds [66,67,68]. Moreover, the changes in the proteome of milk during the extent of a lactation period have also been reported [69,70,71].

Nevertheless, some other studies have used proteomics analysis with the aim of investigating the effects of varying nutritional regimes on the milk composition [72,73] or the effects of undernutrition on milk production [74] or aiming to reveal differences in protein expression between animals in accordance with their milk yield [75]. Finally, a small number of studies have also evaluated the milk proteome of ewes and does, in order to assess potential benefits for consumer health [76,77].

### 4.2. Meat Production

Publications relevant to meat production were among the first ones to report the use of proteomics technologies in sheep or goats [78,79]. Again, most of these studies referred to profiling the proteome of the musculature of the animals, with the aim to describe in detail the proteome of the muscle of these animals [78,79,80,81,82]. The two muscles used primarily for these studies were *M. longissimus* and *M. semitendinosus*, and, notably, differences were found between the various muscles studied in proteomics assessments [83].

Several studies have reported the effects of nutritional management on the muscle proteome of sheep and goats [84,85]. Moreover, the sequential post-mortem changes in these muscles, which are responsible for the taste and texture of meat, were also assessed by means of proteomics methodologies [86,87]. In this respect, the effects of transportation and of the slaughter method on the proteomes of the muscles have also been reported in studies, which, with this approach, have associated animal welfare with the proteome of the meat produced by the animals [88,89,90].

### 4.3. Mastitis

The studies presenting proteomics work in mammary infections of small ruminants can be categorized into those that studied the pathogenesis of the problem and those that studied the early and accurate diagnosis of mastitis.

Research into the pathogenesis of intramammary infections by employing proteomics technologies include the works (a) of Addis et al. [91], who investigated mammary infection with *Mycoplasma agalactiae* (i.e., the mammary form of contagious agalactia), (b) of Addis et al. [92] and Pisanu et al. [93], who investigated mastitis caused by *Streptococcus uberis* (a pathogen of importance primarily in machine-milked flocks and herds [94]) and (c) of Katsafadou et al. [95], who studied mastitis caused by *Mannheimia haemolytica* (the main causal agent of mastitis in meat-producing farms [96]). An important finding of those studies was the significant involvement of the mammary epithelial cells in the process against the organisms causing the infections. Further, the findings have also suggested that the mobility and functionality of neutrophil cells within the mammary gland might be regulated by proteins involved in cell communications [95].

Moreover, proteomics studies have resulted in the identification of biomarkers for the early diagnosis of mastitis in sheep and goats. Addis et al. [92,97] were the first to report that during early mammary infection, cathelicidin proteins were produced by mammary epithelial cells. Subsequently, Katsafadou et al. [98,99] and Bourganou et al. [100] reported the detection of cathelicidins significantly earlier than the rise in somatic cell counts in the milk of ewes and does, respectively. The validity of these findings for the diagnosis of mastitis was confirmed by Cubeddu et al. [101], who corroborated the proteomics findings by using immunochemical techniques. Chiaradia et al. [102] have concluded that, at the early stage of the disease, the detection of proteins in the milk of infected animals potentially can be of better diagnostic value than somatic cell counting. Notably, based on the results of the proteomics works, an ELISA was developed for the detection of cathelicidins in the milk of ewes [103]. This detection of biomarkers (cathelicidin-1 in ewes, cathelicidin-1, and cathelicidin-2 in does) can facilitate early diagnosis of mastitis and can contribute to improved treatment of the infection through the early instigation of appropriate antibiotic administration [104]. Indeed, a significant advantage of using the detection of cathelicidin in the diagnosis of mastitis is that a ‘positive’/‘negative’ result would suffice, without the need for establishing a threshold, as for somatic cell counts.

### 4.4. Gastrointestinal Helminth Infections

Due to the grazing practice of sheep and goats, gastrointestinal nematode infections are important health problems for these species. Administration of anthelmintics is a constituent part of control programs against these infections, but nowadays, resistance against these drugs has been reported worldwide [23,105,106,107,108,109]. Consequently, adjunct approaches have been evaluated to minimize the ensuing difficulties in the control of the infections. In this respect, efforts to improve the immune response of sheep and goats against the infecting helminths contribute to the sustainable control of parasitic infections. Such studies have been based on the following two principles: (i) the genetic selection of ‘responder’ lines of host animals or (ii) the manipulation of nutrition to increase the immune response and ultimately the resistance of these animals to parasitic infections [110,111,112]. Proteomics has been employed in the study for the identification of possible biomarkers of resistant helminths, as well as for the detection of genes and pathways that regulate the development of anthelmintic resistance [113].

In sheep and goats, the immune response to gastrointestinal nematodes is a complex phenomenon that involves many genes [114,115]. Proteomics studies can help to reveal the expression of these genes, thus subsequently helping in the development of the above strategies. For example, the application of proteomics methodologies could help towards elucidation of mechanisms associated with infection by *Haemonchus contortus*, ultimately leading to characterization of breeds and animals as susceptible or resistant to the parasite [115,116].

Proteomics studies have facilitated research on host–parasite–nutrient interactions, especially at the animal gastrointestinal mucosa, which is the parasite–host interface. Findings from high-throughput methodologies have identified molecules excreted from epithelial cells, which can play a role in the expulsion of helminths. For example, the study of the proteome of the intestinal mucosa of animals infected with trichostrongylids has revealed, among others, the upregulation of intelectin, a galactose-binding lectin, which facilitates expulsion of helminths, possibly related to innate immune responses [54,117]. Through performing proteomic and transcriptomic analysis, it was shown that galectins can modulate signaling cascades [118], and moreover, they can inhibit cytokine mRNA transcription and apoptosis in blood mononuclear cells [119]. In vitro studies also revealed that proteomics analysis of viable mucosal intestinal tissue can help in the investigation of specific effector mechanisms, for example, the immune parasite exclusion [120]. This model can help to monitor protein expression patterns during infections of sheep by trichostrongylid parasites.

The immune signatures of sheep infected with *Fasciola hepatica* were analyzed by means of proteomics analysis of peritoneal fluid. Infection-related proteins included proteins from the liver extracellular matrix, as well as immune system proteins, which mediated leucocyte infiltration [121]. The findings provided novel evidence regarding the pathogenetic mechanisms involved in acute *F. hepatica* infection in sheep. Those results were further enhanced by subsequent relevant work; proteomic analysis of the trematode’s excretory and secretory products was performed in cases of long-standing infection serum samples [122]. These more recent findings indicated the central role played by excretory-secretory products in the response of the animals and the protection against *F. hepatica* and also provided further evidence regarding the interactions between the parasite and the host. The entirety of findings can lead to improved diagnosis of the parasitic infection, as well as better recognition of parasite-host interactions, which thus may contribute to better control of the problem.

### 4.5. Zoonotic Relevance of Findings

Proteomics findings in small ruminants may also have some implications for relevant infections in humans, as pathogens causing some of the infections studied have a zoonotic significance.

Proteomics studies, in which immunodominant membrane proteins and virulence factors of *Brucella* spp. or *Campylobacter jejuni* infections in sheep and goats have been identified [123,124], can contribute to the cross-species disease surveillance and diagnostics for the infections in people. Similarly, in fasciolosis, proteomics analyses of excretory-secretory proteins of the causal trematode helminth [121,125] have revealed host–pathogen interaction mechanisms. These can be significant for human infections by the parasite, that way contributing to the discovery of potential translational biomarkers.

Moreover, proteomics analysis of milk from sheep and goats can offer insights into the safety and quality of respective dairy products, which directly connect to food safety and human nutrition. The profiling of milk proteins, including the detection of bioactive peptides, highlights the contribution of proteomics research to the production of safe food.

All the above are specific examples illustrating how proteomics studies in small ruminants can support the One Health paradigm.

### 4.6. Reproductive Management

The majority of original articles related to sheep/goat reproduction referred to profiling the proteomes of various tissues of the genital system. These included ovaries [126], follicles and follicular fluid [127,128] and uterus and uterine luminal fluid [129,130] in non-pregnant females, as well as caruncles and cotyledons [131,132] in pregnant females; they also included testes and accessory genital glands [133,134,135] and semen, seminal fluid, and spermatozoa [136,137,138] in rams and bucks.

In fewer studies, proteomics technologies were used for the evaluation of the fecundity of ewes and does, which is of particular importance in meat production systems, as it relates to the number of lambs or kids born by pregnant females and thus subsequently born. Those studies evaluated protein expression patterns along the hypothalamus-pituitary-ovary axis of ewes and does; their general aim was to detect patterns, based on which animals with high prolificacy could be identified and selected for breeding [139,140,141,142,143]. Also, Hitit et al. [144] have reported the detection of proteins, which could be of use as biomarkers for confirming the fertilizing ability of ram semen. Other authors have studied the effects of nutrition on the reproductive ability of non-pregnant or pregnant ewes or does [145,146], as well as on the fertilizing ability of semen of rams and bucks [133,147], by monitoring the expression of proteins under the various nutritional regimes and applications.

Moreover, proteomics methodologies have been used to assess the fertilizing ability of cryopreserved semen of rams and bucks, by evaluating proteins therein before (i.e., immediately after collection) and after preservation [148,149,150]. The technologies have also been employed to assess the effects of in vitro maturation of cumulus–oocyte complexes of ewes on their subsequent fertilizing ability [151].

Finally, the use of proteomics technologies for the detection of biomarkers related to pregnancy of ewes has been investigated, with the aim of providing an accurate method for pregnancy identification at the early stages, specifically immediately after zygote implantation into the uterus [152,153].

## 5. Conclusions

The results have indicated that the use of proteomics methodological approaches and technologies in sheep and goat work has advanced our knowledge and understanding of the biology of these two animal species in a multitude of fields and topics internationally, with an increasing dissemination and applicability. Through the study of protein expression by means of these methodologies, it has become possible to improve production practices, as well as to elucidate disease mechanisms. All these have been reflected in higher production yields in sheep and goat farms, in improved diagnosis of diseases, and in the implementation of better control schemes against infections. Clearly, the technologies can offer significant support to scientists and clinicians, resulting in improved health management in sheep and goat farms within the context of ‘One-Health’.

## Figures and Tables

**Table 1:** Countries with the most (>10) published papers with sheep or goat work and proteomics, and respective numbers of published papers.

Country ^1^	Total	with Sheep Work	with Goat Work	Total with First Author
CHN	211	98 (46.4% ^2^)	125 (59.2% ^2^)	206 (97.6% ^2^)
USA	50	31 (62.0%)	24 (48.0%)	22 (44.0%)
ITA	49	29 (59.2%)	23 (46.9%)	39 (79.6%)
FRA	39	31 (79.5%)	15 (38.5%)	22 (56.4%)
AUS	38	37 (97.4%)	6 (15.8%)	24 (63.2%)
GBR	32	32 (100.0%)	5 (15.6%)	22 (68.8%)
BRA	26	12 (46.2%)	15 (57.7%)	24 (92.3%)
ESP	22	17 (77.3%)	7 (31.8%)	13 (59.1%)
IND	20	8 (40.0%)	15 (75.0%)	18 (90.0%)
PRT	19	16 (84.2%)	9 (47.4%)	10 (52.6%)
NZL	13	12 (92.3%)	2 (15.4%)	11 (84.6%)
GRC	12	9 (75.0%)	4 (33.3%)	12 (100.0%)
DEU	12	8 (66.7%)	6 (50.0%)	8 (66.7%)

^1^ AUS, Australia; BRA, Brazil; CHN, China; DEU, Germany; GRC, Greece; ESP, Spain; FRA, France; GBR, United Kingdom; IND, India; ITA, Italy; NZL, New Zealand; PRT, Portugal; USA, United States of America. ^2^ Proportion among total papers from the country.

**Table 2:** Scientific establishments with the most (>10) published papers with sheep or goat work and proteomics, and respective numbers of published papers.

Scientific Establishment	Country	Total No. of Published Papers
Chinese Academy of Agricultural Sciences	China	44
Ministry of Agriculture and Rural Affairs	China	40
National Institute for Research in Agriculture, Food, and Environment	France	37
Northwest A&F University—China	China	22
China Agricultural University	China	20
Federal University of Ceara	Brazil	15
Nanjing Agricultural University	China	14
Porto Conte Research	Italy	14
University of Sassari	Italy	14
Inner Mongolia Agricultural University	China	13
Anhui Academy of Agricultural Sciences	China	12
Gansu Agricultural University	China	12
University of Paris Saclay	France	12
Anhui Agricultural University	China	11
Chinese Academy of Sciences	China	11
National Centre for Scientific Research	France	11
University of Milan	Italy	11

**Table 3:** Proteomics methodological approaches and technologies in original articles with sheep or goat work and proteomics, and the respective numbers of original articles in which these were used, and the median year of their publication.

Methodological Approach and Technology ^1^	No. of Original Articles	Median Year of Publication
2-DE/SDS-PAGE	13 (2.9%)	2018 (IQR ^2^: 5.5 years)
2-DE and LC-MS/MS	50 (11.2%)	2018 (IQR: 6 years)
2-DE and MALDI-TOF MS	57 (12.7%)	2015 (IQR: 8 years)
2-DE and MALDI-TOF MS and LC-MS/MS	9 (2.0%)	2011 (IQR: 4 years)
in silico analysis	7 (1.6%)	2021 (IQR: 7.5 years)
GeLC-MS/MS	53 (11.8%)	2020 (IQR: 7 years)
LC-MS/MS	237 (52.9%)	2022 (IQR: 4 years)
LC-MS/MS and MALDI-TOF MS	5 (1.1%)	2019 (IQR: 7 years)
MALDI/SELDI-TOF MS	8 (1.8%)	2010 (IQR: 5 years)
Protein microarrays	2 (0.4%)	2021 (IQR: 2.5 years)
SDS-PAGE and MALDI-TOF MS	7 (1.6%)	2012 (IQR: 7 years)

^1^ 2-DE, two-dimensional gel electrophoresis; SDS-PAGE, sodium dodecyl–sulfate polyacrylamide gel electrophoresis; LC-MS/MS, liquid chromatography–tandem mass spectrometry; MALDI-TOF MS, matrix-assisted laser desorption/ionization coupled to time-of-flight mass spectrometry; GeLC-MS/MS, polyacrylamide gel electrophoresis followed by liquid chromatography–tandem mass spectrometry SELDI-TOF MS: Surface-enhanced laser desorption/ionization time-of-flight mass spectrometry. ^2^ Interquartile range.

**Table 4:** Keywords featured in >10 published papers with sheep or goat work and proteomics, and respective numbers of published papers in which they appeared.

Keyword	No. of Published Papers
proteomics	135
sheep	80
proteome	46
goat	39
mass spectrometry	29
2-DE	28
ovine	20
isobaric tag for relative and absolute quantitation (iTRAQ)	19
biomarker	18
proteomic	18
goat milk	16
milk	15
mastitis	14
colostrum	13
protein	13
metabolomics	12
whey proteins	11

**Table 5:** Journals where most papers with sheep or goat work and proteomics were published (from countries with the most relevant papers), and the respective numbers of published papers.

Country	Journal(s)	No. of Published Papers
Australia	*Journal of Proteomics*	7
Brazil	*Reproduction in Domestic Animals*	4
China	*Journal of Proteomics*	16
France	*Journal of Dairy Science*, *Journal of Proteomics*, *Molecular Biosystems*, *Proteomics*	3
Germany	*Analytical and Bioanalytical Chemistry*	2
Greece	*Animals*, *Journal of Proteomics*, *Pathogens*	2
India	no journal with >1 published papers	n/a
Italy	*Journal of Proteomics*	7
New Zealand	*Journal of Proteomics*	3
Portugal	*Journal of Proteomics*, *Plos One*	3
Spain	*Scientific Reports*	2
United Kingdom	*Proteomics*	4
United States of America	*Biology of Reproduction*, *Journal of Dairy Science*	3

**Table 6:** Journals where most papers with sheep or goat work and proteomics were published (in accordance with the topic of study), and the respective numbers of published papers.

Topic of Study	Journal(s)	No. of Published Papers
animal disease	*Journal of Proteomics*	10
models for the study of conditions in humans	*International Journal of Molecular Sciences*	2
physiology	*International Journal of Molecular Sciences*, *Journal of Dairy Science*, *Journal of Proteomics*, *Plos One*, *Proteomics*	4
sheep/goat production	*Journal of Proteomics*	14
sheep/goat reproduction	*Journal of Proteomics*	9

**Table 7:** Results of multivariable analysis for predictors associated with the number of annual citations in original articles with sheep or goat work and proteomics.

Variables	Relative Risk (±s.e. ^1^)	*p*
Number of References Cited in the Paper		<0.0001
per unit increase	1.027 ± 1.006	<0.0001
Topic of Study		0.015
animal diseases (1.5 (2.0) ^2^)	reference	--
small ruminants as models for the study of various conditions in humans (1.7 (2.2))	1.495 ± 1.784	0.12
physiology (1.7 (3.1))	1.560 ± 1.250	0.048
sheep/goat production (2.5 (3.0))	1.638 ± 1.137	0.0002
sheep/goat reproduction (1.8 (2.9))	1.222 ± 1.113	0.06
Animal Species Involved in the Study		0.044
sheep (1.6 (2.7))	reference	-
goats (2.0 (3.1))	1.708 ± 1.371	0.09
both species (2.6 (2.0))	1.422 ± 1.340	0.23

^1^ Standard error. ^2^ Median (interquartile range).

**Table 8:** Eigenvalues for principal component analysis for the number of annual citations in original articles with sheep or goat work and proteomics.

Parameter	PC1	PC2	PC3	PC4	PC5
Eigenvalue	1.39	1.08	0.96	0.81	0.76
% of Variance	27.9	21.5	19.2	16.2	15.1
Cumulative variance (%)	27.9	49.4	68.6	84.9	100.0

## Data Availability

All data are available on the Web of Science platform and in the Supplementary Materials.

## Supplementary Materials

The following supporting information can be downloaded at: https://www.mdpi.com/article/10.3390/ani15203050/s1, Table S1: PRISMA flow diagram for the identification and exclusion of records from Web of Science database; Table S2: Details of multivariable models employed for the evaluation of potential associations with the number of annual citations in original articles with sheep or goat work and proteomics; Figure S1: Box-and-whisker plot of year of publication of original articles or reviews with sheep or goat work and proteomics; Figure S2: Scatter plot of number of published papers with sheep or goat work and proteomics in accord with year of publication; Table S3: Countries with published papers with sheep or goat work and proteomics and respective numbers of published papers and published papers with first author from that country; Table S4: Median year of publication of papers with sheep or goat work and proteomics, in accord with the country of origin; Table S5: Countries with scientific establishments with at least three published papers with sheep or goat work and proteomics and respective numbers of scientific establishments; Figure S3: Scatter plot of the number of scientific establishments in a country and the number of published papers that originated from that country; Table S6: Number of original articles with sheep or goat work and proteomics, in accord with the type of work described therein and with the country of origin; Figure S4: Scatter plot of the proportion of original articles presenting experimental work with sheep or goat work and proteomics, in accord with year of publication; Table S7: Number of original articles with sheep or goat work and proteomics, in accord with the topic of study described therein and with the animal species referred to in respective studies; Table S8: Proportion of original articles with sheep or goat work and proteomics, in accord with the type of work and the topic of study described therein; Table S9: Number of original articles with sheep or goat work and proteomics, in accord with the disease described therein; Table S10: Number of original articles with sheep or goat work and proteomics, in accord with conditions in humans studied by using sheep or goats as models; Table S11: Number of original articles with sheep or goat work and proteomics, in accord with the topic of study described therein and with the country of origin; Figure S5: Bar plot of the proportion of original articles with affiliation of veterinary educational establishments, in accord with the topic of study; Table S12: Number of original articles with sheep or goat work and proteomics, in accord with the particular aspect within the general field of sheep/goat production described therein and with the animal species referred to in respective studies; Table S13: Number of original articles with sheep or goat work and proteomics, in accord with the tissue analyzed in the respective studies; Table S14: Tissues analyzed in studies in original articles with sheep or goat work and proteomics, in accord with animal species referred to in respective study; Figure S6: Box-and-whisker plot of year of publication of original articles with sheep or goat work and proteomics, in accord with the proteomics methodological approaches and technologies used; Table S15: Proteomics methodological approaches and technologies in original articles with sheep or goat work and proteomics, in accord with the type of work described therein; Table S16: Proteomics technologies and methodological approaches in original articles with sheep or goat work and proteomics, in accord with the topic of study; Table S17: Proteomics methodological approaches and technologies in original articles with sheep or goat work and proteomics, in accord with the tissue analyzed; Table S18: Keywords featured in > 3 published papers with sheep or goat work and proteomics; Figure S7: Word cloud of the most frequently occurring keywords in published papers with sheep or goat work and proteomics; Figure S8: Scatter plot of the number of keywords per published papers with sheep or goat work and proteomics in accord with year of publication; Table S19: Journals with published papers with sheep or goat work and proteomics; Figure S9: Box-and-whisker plot of year of publication of papers in the five journals with most papers and content on proteomics–molecular studies and in the eight journals with most papers and content on clinical–health management studies; Figure S10: Scatter plot of the number of authors per published papers with sheep or goat work and proteomics in accord with year of publication; Table S20: Number of authors in published papers with sheep or goat work and proteomics, in accord with the country of origin; Table S21: Number of authors in original articles with sheep or goat work and proteomics, in accord with the topic of study; Table S22: Number of cited references in original articles with sheep or goat work and proteomics, in accord with the type of work; Table S23: Number of cited references in original articles with sheep or goat work and proteomics, in accord with the topic of study; Table S24: Results of univariable analyses for predictors associated with the number of annual citations in original articles with sheep or goat work and proteomics; Table S25: Results of univariable analyses for predictors associated with the number of annual citations in reviews with sheep or goat work and proteomics; Figure S11: Scree plot for principal component analysis for the number of annual citations in original articles with sheep or goat work and proteomics.
