# Supplementary material for: Appraisal of the Use of Proteomics Methodological Approaches and Technologies on Sheep and Goat Research and Clinical Work
Source: Animals (Basel). 2025 Oct 20;15(20):3050. doi: 10.3390/ani15203050 (PMC12562089; doi:10.3390/ani15203050)

Appraisal of the Use of Proteomics Methodological Approaches and Technologies in the Health Management of Sheep and Goats

Maria V. Bourganou †, Georgia A. Vaitsi †, Dimitra V. Liagka, Charalambia C. Michael, Eleni I. Katsarou, Dimitris C. Chatzopoulos, Daphne T. Lianou, Natalia G. C. Vasileiou, George Th. Tsangaris, Vasia S. Mavrogianni, George C. Fthenakis, ‡ and Angeliki I. Katsafadou ‡

Table S1. PRISMA flow diagram for the identification and exclusion of records from Web of Science database [1,2]

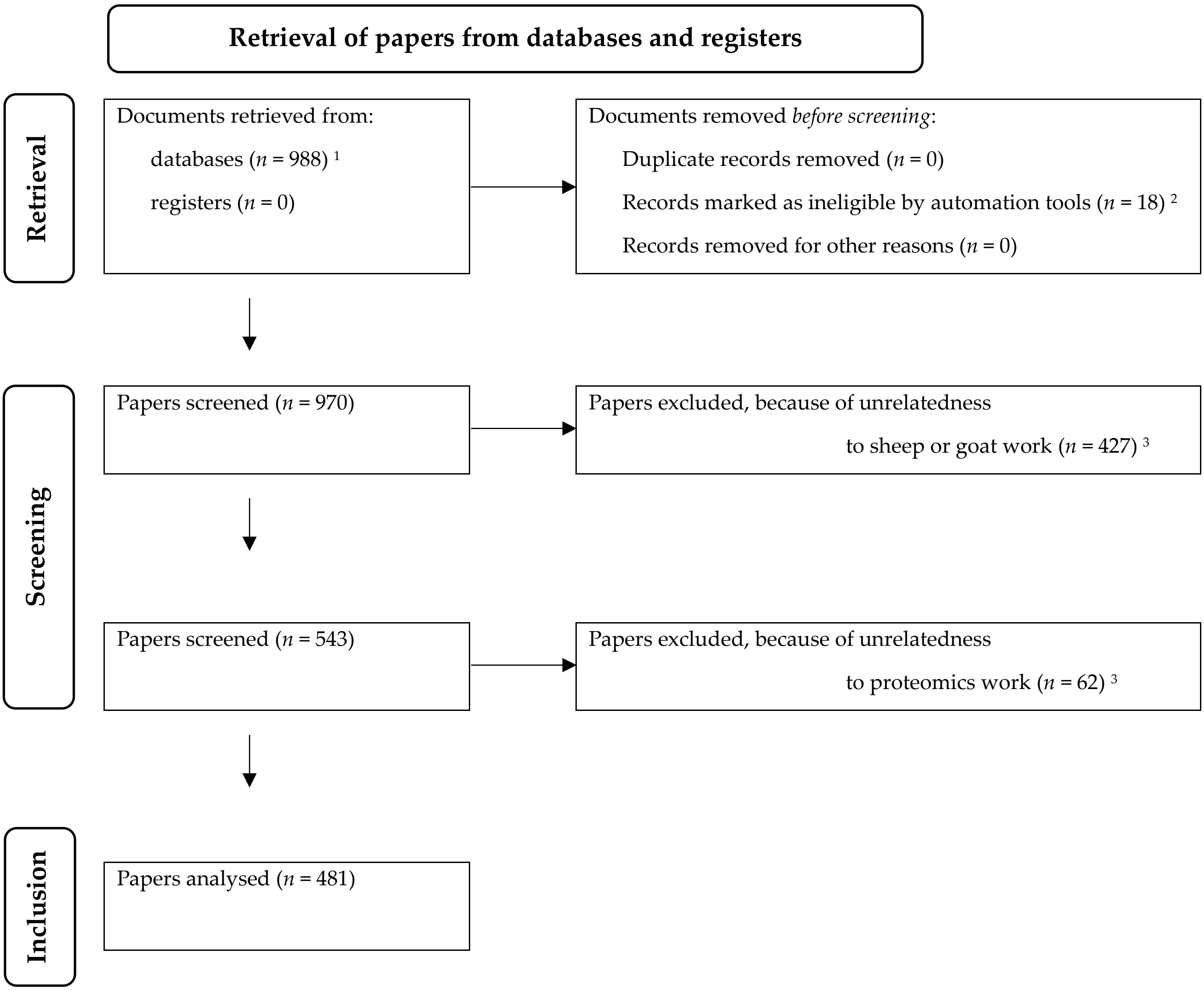

[1] Search performed in Web of Science database.
[2] Documents other than 'Articles' or Review articles' excluded – exclusion performed by the Web of Science platform.
[3] Exclusion performed through assessment of papers by authors.

References

1. Page, M.J.; McKenzie, J.E.; Bossuyt, P.M.; Boutron, I.; Hoffmann, T.C.; Mulrow, C.D. Shamseer, L.S.; Tetzlaff, J.M.; *et al.* The PRISMA 2020 statement: an updated guideline for reporting systematic reviews. *Br. Med. J.* **2021**, *372*, n71.
2. PRISMA Flow Diagram. https://www.prisma-statement.org/prisma-2020-flow-diagram (accessed on 16 October 2024).

Table S2. Details of multivariable models (n = 2) employed for the evaluation of potential associations with the number of annual citations in original articles with sheep or goat work and proteomics.

Outcome	Variables		
	assessed in uni-variable analyses (n)	offered to the multi-variable models (n)	required in the final models
citations received by an original article yearly	13	7	(a) animal species referred to in papers, (b) country of first author, (c) field of study, (d) Proteomics methodological approaches and technologies used, (e) No. of cited references
citations received by a review yearly	8	1	(a) No. of cited references

Figure S1. Box-and-whisker plot of year of publication of original articles (light blue; *n* = 448) or reviews (light green; *n* = 33) with sheep or goat work and proteomics.

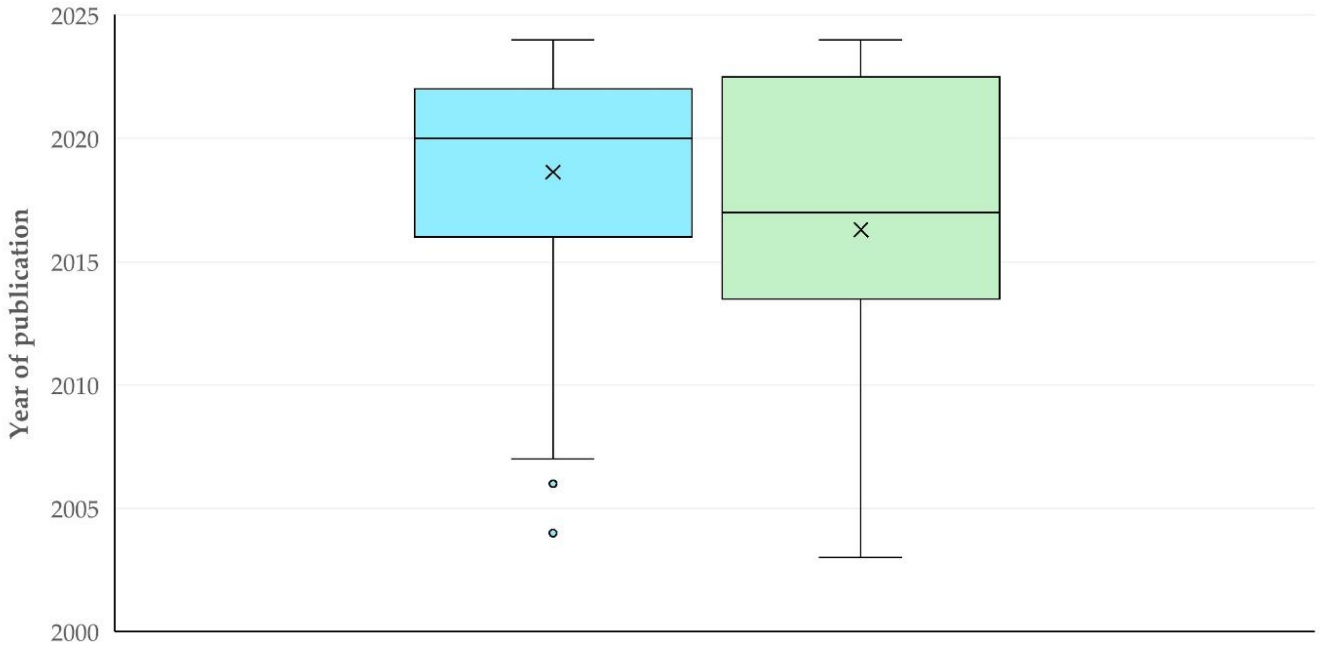

Figure S2. Scatter plot of number of published papers with sheep (green; n = 299) or goat (brown; n = 228) work and proteomics in accord with year of publication (dashed lines are trendlines).

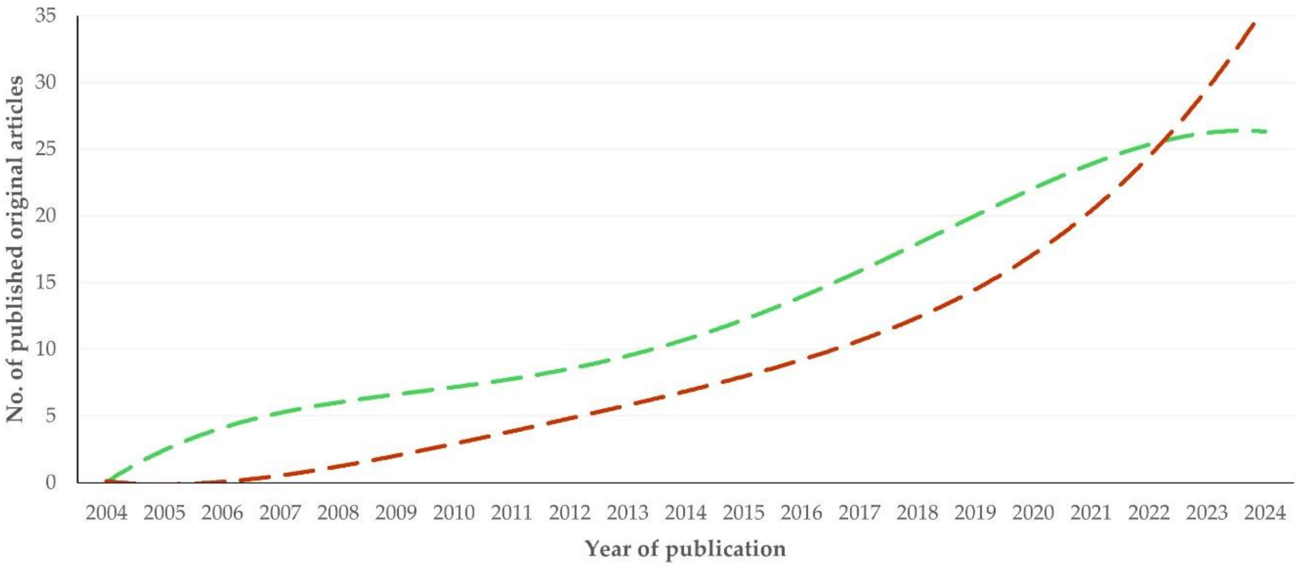

Table S3. Countries with published papers with sheep or goat work and proteomics and respective numbers of published papers and published papers with first author from that country.

Country	Number of papers with origin from the country	
	Total	Total with first author
China	211	206 (97.6% [1])
USA	50	22 (44.0%)
Italy	49	39 (79.6%)
France	39	22 (56.4%)
Australia	38	24 (63.2%)
United Kingdom	32	22 (68.8%)
Brazil	26	24 (92.3%)
Spain	22	13 (59.1%)
India	20	18 (90.0%)
Portugal	19	10 (52.6%)
New Zealand	13	11 (84.6%)
Greece	12	12 (100.0%)
Germany	12	8 (66.7%)
Ireland	10	4 (40.0%)
Switzerland	9	2 (22.2%)
Egypt	9	1 (11.1%)
St Kitts & Nevis	7	2 (28.6%)
Austria	6	6 (100.0%)
Malaysia	6	6 (100.0%)
Canada	6	1 (16.7%)
Denmark	6	1 (16.7%)
Pakistan	6	0 (0.0%)
Poland	5	4 (80.0%)
Czechia	5	3 (60.0%)
Iran	5	2 (40.0%)
Belgium	4	0 (0.0%)
Luxembourg	4	0 (0.0%)
Algeria	3	2 (66.7%)
Slovakia	3	1 (33.3%)
Morocco	3	0 (0.0%)
The Netherlands	3	0 (0.0%)
Uruguay	3	0 (0.0%)
Norway	2	2 (100.0%)
Israel	2	1 (50.0%)
Saudi Arabia	2	1 (50.0%)
Taiwan	2	1 (50.0%)
Tunisia	2	1 (50.0%)
Turkiye	2	1 (50.0%)
Bulgaria	2	0 (0.0%)
Albania	1	1 (100.0%)
Finland	1	1 (100.0%)
Hungary	1	1 (100.0%)
Indonesia	1	1 (100.0%)
Japan	1	1 (100.0%)
Mexico	1	1 (100.0%)
South Africa	1	1 (100.0%)
Venezuela	1	1 (100.0%)
Argentina	1	0 (0.0%)
Croatia	1	0 (0.0%)
Equador	1	0 (0.0%)
Nigeria	1	0 (0.0%)
Singapore	1	0 (0.0%)

South Korea	1	0 (0.0%)
Sudan	1	0 (0.0%)
Sweden	1	0 (0.0%)
Thailand	1	0 (0.0%)
Yemen	1	0 (0.0%)

[1] Proportion among total papers from the country.

Table S4. Median year of publication of papers with sheep or goat work and proteomics, in accord with the country of origin [1].

Countries	Median year of publication of papers (interquartile range)
Australia	2014 (8 years)
Brazil	2019 (4 years)
China	2022 (4 years)
France	2015 (8 years)
Germany	2016 (6 years)
Greece	2021 (4 years)
India	2021 (5 years)
Italy	2017 (7.5 years)
New Zealand	2018 (6.5 years)
Portugal	2014 (4.5 years)
Spain	2021 (6 years)
United Kingdom	2011 (8 years)
United States of America	2016 (6 years)

[1] Only countries with most (> 10) published papers considered.

Table S5. Countries with scientific establishments with at least three published papers with sheep or goat work and proteomics and respective numbers of scientific establishments.

Country	Number of scientific establishments with ≥ 3 published papers per establishment
China	28
France	11
United Kingdom	10
Australia	8
Italy	7
United States of America	7
Brazil	6
Portugal	5
Spain	4
Greece	3
Germany	2
India	2
New Zealand	2
Algeria	1
Austria	1
Denmark	1
Ireland	1
Luxembourg	1
Malaysia	1
Morocco	1
Poland	1
Switzerland	1
Turkey	1
Uruguay	1

Figure S3. Scatter plot of the number of scientific establishments (with ≥ 3 published papers) in a country and the number of published papers that originated from that country.

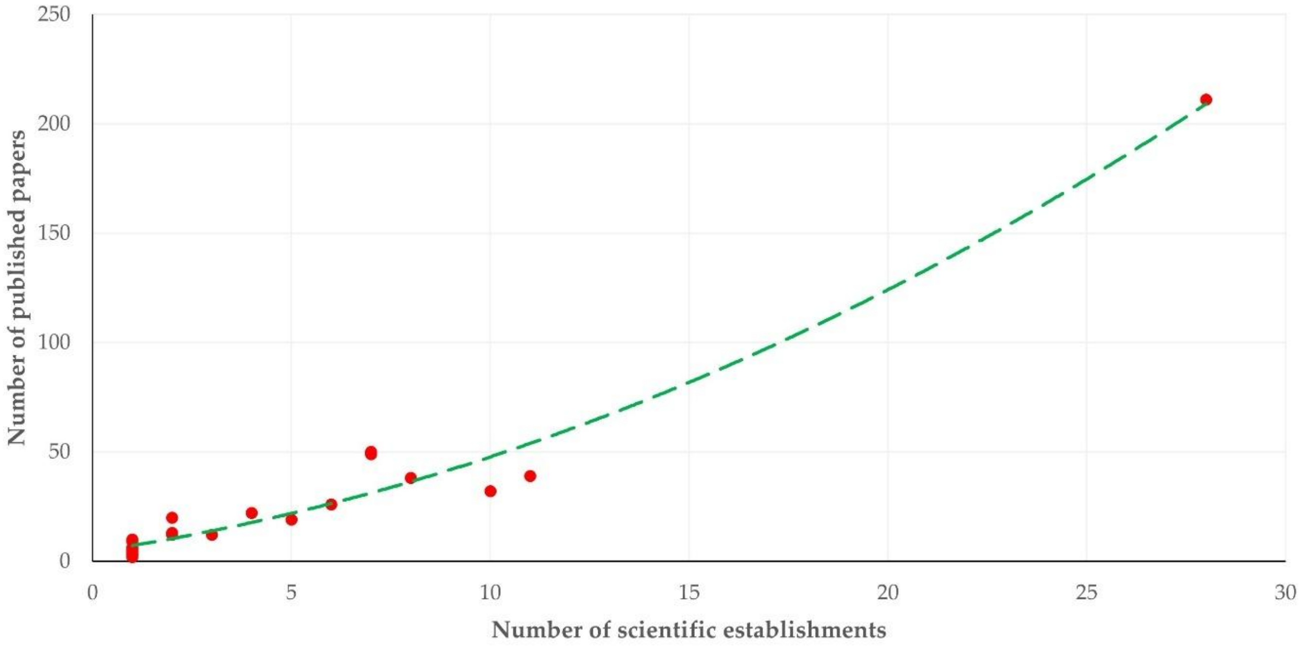

Table S6. Number of original articles with sheep or goat work and proteomics, in accord with the type of work described therein and with the country of origin [1].

	Type of work			
Country	Field	Experimental	*In vitro*	Computational
Australia	3	30	0	0
Brazil	6	20	0	0
China	15	187	2	2
France	6	29	0	2
Germany	0	11	0	0
Greece	1	5	1	2
India	2	16	0	0
Italy	13	32	0	0
New Zealand	3	9	0	0
Portugal	2	15	0	0
Spain	2	20	0	0
United Kingdom	3	24	0	0
United States of America	2	44	0	0

[1] Only countries with most (> 10) published papers considered.

Figure S4. Scatter plot of the proportion of original articles presenting experimental work with sheep or goat work and proteomics, in accord with year of publication (dashed lines are trendlines [1]).

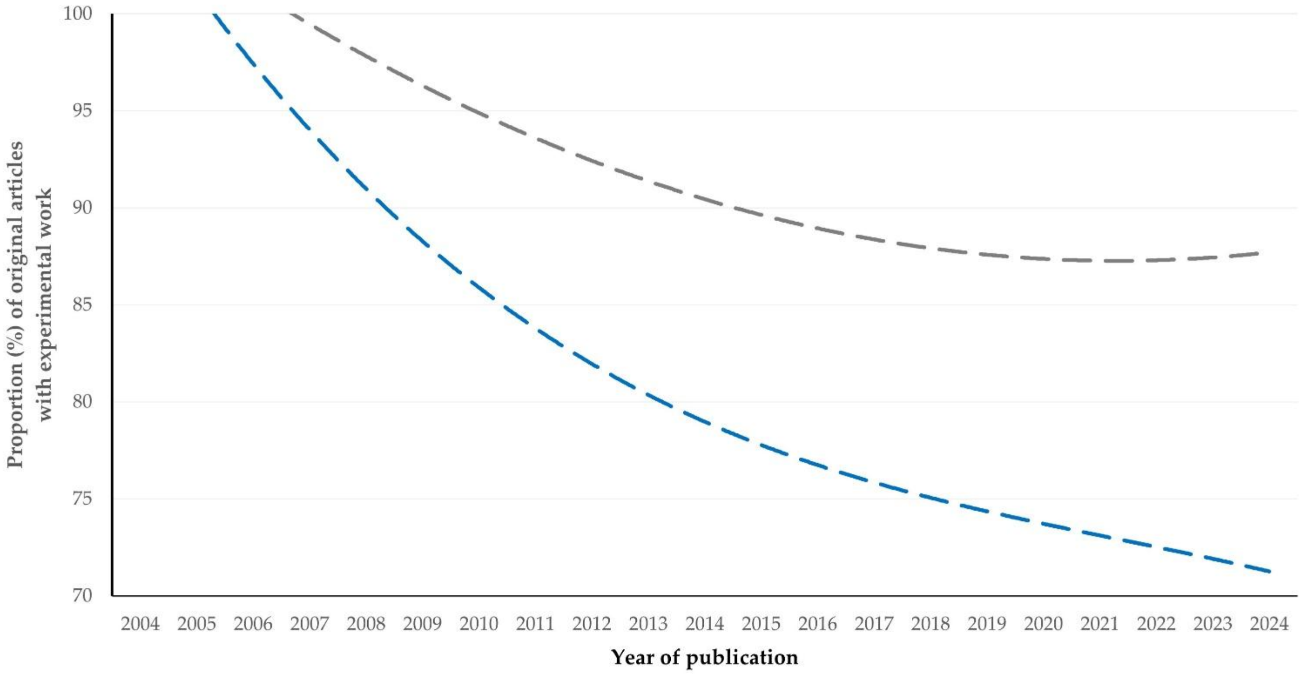

[1] blue: articles from countries in the European Union, grey: articles from all other countries of the world.

Table S7. Number of original articles with sheep or goat work and proteomics, in accord with the topic of study described therein and with the animal species referred to in respective studies.

	Number of original articles referring to	
Topic of study	sheep	goats
Animal diseases	60 (22.1% [1])	29 (13.9% [1])
Use of small ruminants as models	23 (8.5%)	0 (0.0%)
Physiology	58 (21.3%)	42 (20.0%)
Sheep / goat production	68 (25.0%)	114 (52.8%)
Sheep / goat reproduction	72 (26.5%)	30 (14.3%)

[1] Proportion of original articles among all referring to the animal species.

Table S8. Proportion of original articles with sheep or goat work and proteomics, in accord with the type of work and the topic of study described therein.

Type of work	Topic of study					
	Animal diseases	Use of small ruminants as models	Physiology	Sheep / goat production	Sheep / goat reproduction	*p*
Field	13.0% [1]	0.0%	13.0%	42.6%	31.5%	< 0.0001
Computational	57.1%	0.0%	28.6%	14.3%	0.0%	0.19
Experimental	17.3%	5.8%	20.6%	35.9%	20.4%	< 0.0001
In vitro	33.3%	0.0%	66.7%	0.0%	0.0%	0.41

[1] Proportion of original articles among all with the particular type of work therein.

Table S9. Number of original articles with sheep or goat work and proteomics, in accord with the health problem described therein.

Health problem	Number of original articles
Mastitis	23
Fasciolosis	6
Haemonchosis	5
Larval metacestodoses	5
Teladorsagiosis	5
Paratuberculosis	4
Peste des petits ruminants	4
Pseudotuberculosis	4
Brucellosis	3
Cadmium toxicosis	2
Ovine pulmonary adenocarcinoma	2
Prion disease	2
Uterine disorder	2
Abortion associated with *Campylobacter* spp.	1
Bluetongue	1
Borrelia infection	1
Caprine arthritis encephalitis	1
Coccidiosis	1
Contagious agalactia	1
Foot-and-mouth disease	1
Meliodosis	1
Musculosceletal disorder	1
Q fever	1
Ruminal acidosis	1
Trichostrongylosis	1
Trypanosomiasis	1
Vaginal prolapse	1

Table S10. Number of original articles with sheep or goat work and proteomics, in accord with conditions in humans studied by using sheep or goats as models.

Conditions studied	Number of original articles
Atrial fibrillation	2
Myocardial infarction	2
Acute decompensated heart failure	1
Acute kidney injury	1
Cartilage injury and regeneration / osteoarthritis	1
Chronic exposure to environmental chemical mixtures	1
Congenital diaphragmatic hernia	1
Congenital heart diseases	1
Copper-associated liver disease	1
Disseminated intravascular coagulation	1
Gastric adenocarcinoma,	1
Huntington's disease	1
Intrauterine growth restriction	1
Leucaemia	1
Lung neuroendocrine tumours	1
Lymphatic muscle remodelling	1
Neuronal ceroid lipofuscinoses	1
Non-alcoholic fatty liver disease – Non-alcoholic steatohepatitis	1
Optic nerve degeneration	1
Osteoarthritis	1
Squamous cell carcinomas	1
Ventricular arrhythmias	1

Table S11. Number of original articles with sheep or goat work and proteomics, in accord with the topic of study described therein and with the country of origin.

Country [1]	Topic of study				
	animal diseases	use of small ruminants as models	physiology	sheep / goat production	sheep / goat reproduction
Australia	9 [2]	3 [2]	8 [2]	6 [2]	7 [2]
Brazil	5	0	2	6	15
China	20	3	41	99	49
France	7	3	7	10	11
Germany	3	1	6	1	0
Greece	6	0	0	2	1
India	4	0	3	9	3
Italy	14	3	7	21	0
New Zealand	2	3	0	6	1
Portugal	1	0	8	8	0
Spain	6	3	4	7	2
United Kingdom	6	5	10	1	5
United States of America	8	5	10	13	11

[1] Only countries with most (> 10) published papers considered.
[2] No. of original articles.

Figure S5. Bar plot of the proportion of original articles with affiliation of veterinary educational establishments (violet: with affiliation of veterinary educational establishments (n = 157), grey: with no affiliation of veterinary educational establishments (n = 291), in accord with the topic of study (full pattern: articles in the general field of animal diseases (n = 81), motif pattern: articles in all other fields (n = 367)).

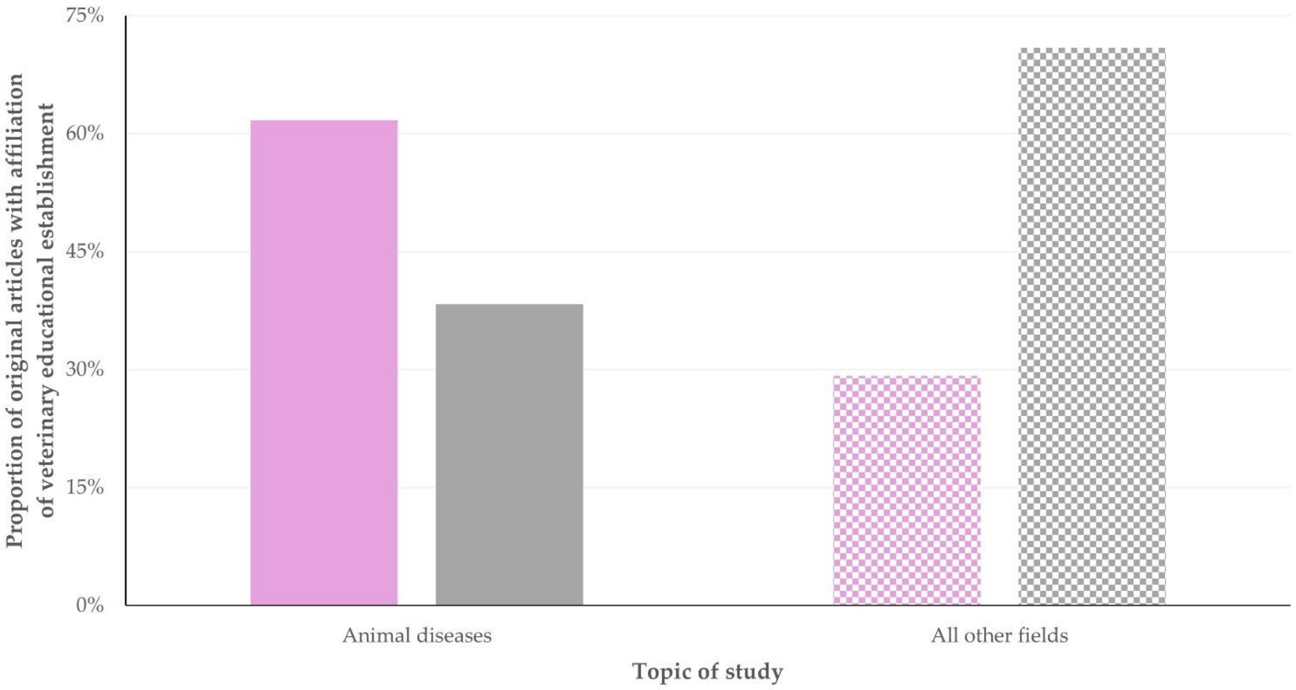

Table S12. Number of original articles with sheep or goat work and proteomics, in accord with the particular aspect within the general field of sheep / goat production described therein and with the animal species referred to in respective studies.

	Number of original articles referring to	
Particular aspect of sheep / goat production	sheep	goats
Hair production	0 (0.0% [1])	19 (16.5% [1])
Horn production	1 (1.5%)	1 (0.9%)
Meat production	27 (39.7%)	17 (14.8%)
Milk production	24 (35.3%)	77 (67.0%)
Wool production	16 (23.5%)	0 (0.0%)

[1] Proportion of original articles among all referring to the respective animal species.

Table S13. Number of original articles with sheep or goat work and proteomics, in accord with the tissue analysed in the respective studies.

Tissue analysed	Number of original articles with relevant work
Milk	81
Blood	46
Muscle	41
Milk fat globule membrane	29
Semen	24
Skin	22
Ovary	21
Liver	20
Uterus	16
Rumen	14
Mammary gland	12
Colostrum	11
Embryo	10
Brain	9
Fibers	9
Intestine	9
Heart	8
Saliva	8
Spermatozoa	8
Abomasum	7
Adipose tissue	7
Lung	7
Testis	6
Bone	5
Cerebrospinal fluid	4
Oviduct	4
Spleen	4
Vaginal excretions	4
Foetal membranes	3
Lymph node	3
Epididymis	2
Horn	2
Kidney	2
Nasal mucus	2
Pancreas	2
Pituitary	2
Urine	2
Wool	2
Accessory genital glands	1
Articular cartilage	1
Bile	1
Bile duct	1
Eye	1
Faeces	1
Lymph	1
Nerves	1
Peritoneal fluid	1
Tears	1
Tendon	1
Tooth	1
Tracheal fluid	1

Table S14. Tissues analysed in studies in original articles with sheep or goat work and proteomics, in accord with animal species referred to in respective study.

Tissue analysed	Number of original articles referring to	
	sheep	goats
Abomasum	6 (2.2%)	0 (0.0%)
Accessory glands	1 (0.4%)	0 (0.0%)
Adipose tissue	5 (1.8%)	3 (1.4%)
Articular cartilage	1 (0.4%)	0 (0.0%)
Bile	1 (0.4%)	1 (0.5%)
Bile duct	1 (0.4%)	1 (0.5%)
Blood	34 (12.5%)	17 (8.1%)
Bone	5 (1.8%)	1 (0.5%)
Brain	6 (2.2%)	3 (1.4%)
Cerebrospinal fluid	4 (1.5%)	4 (1.9%)
Colostrum	3 (1.1%)	9 (4.3%)
Embryo	9 (3.3%)	1 (0.5%)
Epididymis	2 (0.7%)	0 (0.0%)
Eye	1 (0.4%)	0 (0.0%)
Faeces	1 (0.4%)	0 (0.0%)
Fibers	5 (1.8%)	7 (3.3%)
Foetal membranes	3 (1.1%)	0 (0.0%)
Heart	7 (2.5%)	1 (0.5%)
Horns	1 (0.4%)	1 (0.5%)
Intestine	7 (2.5%)	1 (0.5%)
Kidney	2 (0.7%)	0 (0.0%)
Liver	13 (4.7%)	7 (3.3%)
Lung	7 (2.5%)	1 (0.5%)
Lymph	1 (0.4%)	0(0.0%)
Lymph node	3 (1.1%)	0 (0.0%)
Mammary gland	4 (1.5%)	8 (3.8%)
Milk	29 (10.7%)	63 (30.0%)
Milk fat globule membrane	7 (2.5%)	23 (11.0%)
Muscle	24 (8.8%)	18 (8.6%)
Nasal mucus	2 (0.7%)	1 (0.5%)
Nerves	1 (0.4%)	0 (0.0%)
Ovary	17 (6.2%)	4 (1.9%)
Oviduct	2 (0.7%)	2 (1.0%)
Pancreas	0 (0.0%)	2 (1.0%)
Peritoneal fluid	1 (0.4%)	1 (0.5%)
Pituitary	1 (0.4%)	1 (0.5%)
Rumen	7 (2.5%)	7 (3.3%)
Saliva	7 (2.5%)	5 (2.4%)
Semen	12 (4.4%)	14 (6.7%)
Skin	9 (3.3%)	13 (6.2%)
Spermatozoa	4 (1.5%)	5 (2.4%)
Spleen	2 (0.7%)	3 (1.4%)
Tear	1 (0.4%)	0 (0.0%)
Tendon	1 (0.4%)	0 (0.0%)
Testis	6 (2.2%)	0 (0.0%)
Tooth	1 (0.4%)	0 (0.0%)
Tracheal fluid	1 (0.4%0	0 (0.0%)
Urine	2 (0.7%)	0 (0.0%)
Uterus	12 (4.4%)	4 (1.9%)
Vaginal excretions	4 (1.5%)	0 (0.0%)
Wool	2 (0.7%)	0 (0.0%)

Figure S6. Box-and-whisker plot of year of publication of original articles with sheep or goat work and proteomics, in accord with the proteomics methodological approaches and technologies used (from left to right: 2-DE / SDS-PAGE (*n* = 12), 2-DE & LC-MS/MS (*n* = 50), 2-DE & MALDI-TOF MS (*n* = 57), 2-DE & MALDI-TOF MS & LC-MS/MS (*n* = 9), *in silico* analysis (*n* = 7), GeLC-MS/MS (*n* = 53), LC-MS/MS (*n* = 237), LC-MS/MS & MALDI-TOF MS (*n* = 5), MALDI / SELDI - TOF MS (*n* = 8), protein microarrays (*n* = 2), SDS-PAGE & MALDI-TOF MS (*n* = 7) [1]).

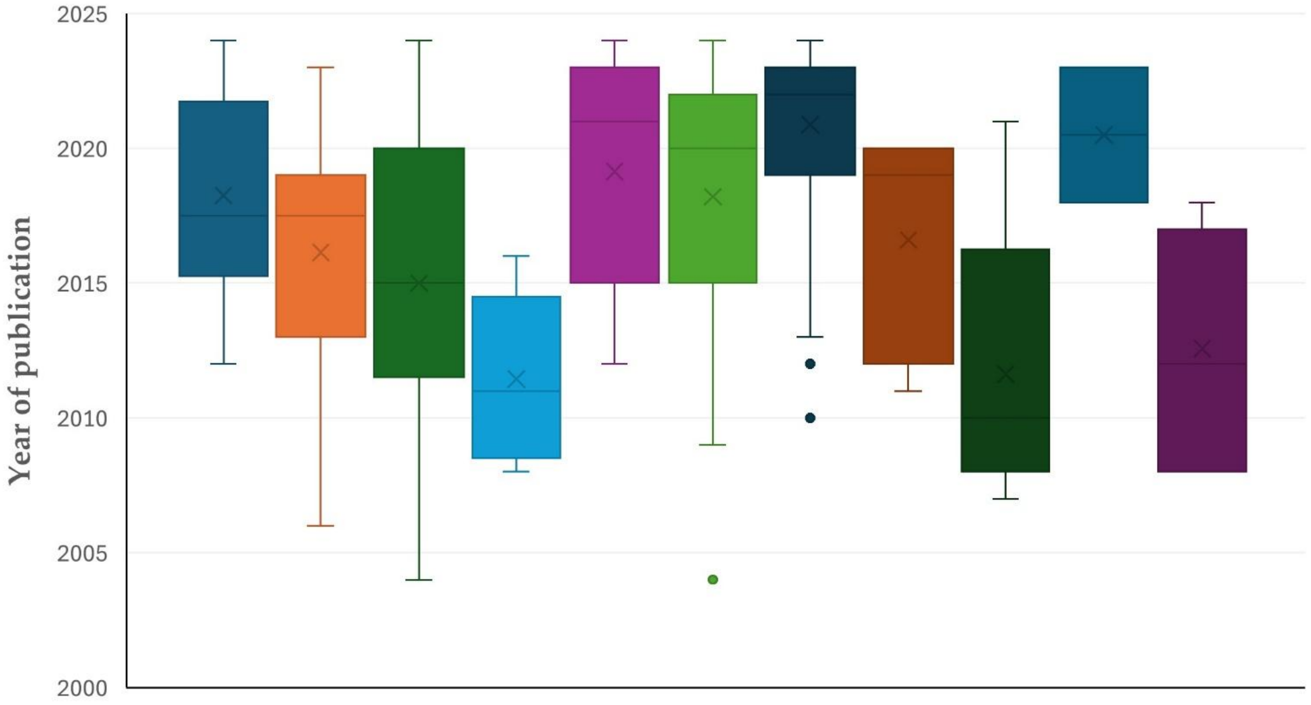

[1] 2-DE: two-dimensional gel electrophoresis, SDS-PAGE: Sodium dodecyl-sulfate polyacrylamide gel electrophoresis, LC-MS/MS: liquid chromatography-tandem mass spectrometry, MALDI-TOF MS: matrix-assisted laser desorption/ionization coupled to time-of-flight mass spectrometry, GeLC-MS/MS: polyacrylamide gel electrophoresis followed by liquid chromatography-tandem mass spectrometry SELDI - TOF MS: Surface-enhanced laser desorption/ionization Time-of-Flight mass spectrometry.

Table S15. Proteomics methodological approaches and technologies in original articles with sheep or goat work and proteomics, in accord with the type of work described therein.

Methodological approaches and technologies [1]	Type of work			
	Field	Experimental	*In vitro*	Computational
2-DE / SDS-PAGE	4 [2]	9 [2]	0 [2]	0 [2]
2-DE & LC-MS/MS	8	42	0	0
2-DE & MALDI-TOF MS	2	55	0	0
2-DE, MALDI-TOF MS, LC-MS/MS	2	7	0	0
in silico analysis	0	0	0	7
GeLC-MS/MS	14	39	0	0
LC-MS/MS	19	215	3	0
LC-MS/MS & MALDI-TOF MS	3	2	0	0
MALDI / SELDI - TOF MS	1	7	0	0
Protein microarrays	0	2	0	0
SDS-PAGE & MALDI-TOF MS	1	6	0	0

[1] 2-DE: two-dimensional gel electrophoresis, SDS-PAGE: Sodium dodecyl-sulfate polyacrylamide gel electrophoresis, LC-MS/MS: liquid chromatography-tandem mass spectrometry, MALDI-TOF MS: matrix-assisted laser desorption/ionization coupled to time-of-flight mass spectrometry, GeLC-MS/MS: polyacrylamide gel electrophoresis followed by liquid chromatography-tandem mass spectrometry SELDI - TOF MS: Surface-enhanced laser desorption/ionization Time-of-Flight mass spectrometry.

[2] No. of original articles.

Table S16. Proteomics methodological approaches and technologies in original articles with sheep or goat work and proteomics, in accord with the topic of study.

Methodological approaches and technologies [1]	Topic of study				
	animal diseases	use of small ruminants as models	physiology	sheep / goat production	sheep / goat reproduction
2-DE / SDS-PAGE	2 [2]	0 [2]	2 [2]	7 [2]	3 [2]
2-DE & LC-MS/MS	13	2	9	13	13
2-DE & MALDI-TOF MS	12	3	10	24	9
2-DE, MALDI-TOF MS, LC-MS/MS	4	0	1	3	1
in silico analysis	4	0	2	1	0
GeLC-MS/MS	9	0	14	22	12
LC-MS/MS	29	16	48	94	58
LC-MS/MS & MALDI-TOF MS	2	1	1	0	1
MALDI / SELDI - TOF MS	4	0	3	1	0
Protein microarrays	1	1	0	0	0
SDS-PAGE & MALDI-TOF MS	1	0	3	2	1

[1] 2-DE: two-dimensional gel electrophoresis, SDS-PAGE: Sodium dodecyl-sulfate polyacrylamide gel electrophoresis, LC-MS/MS: liquid chromatography-tandem mass spectrometry, MALDI-TOF MS: matrix-assisted laser desorption/ionization coupled to time-of-flight mass spectrometry, GeLC-MS/MS: polyacrylamide gel electrophoresis followed by liquid chromatography-tandem mass spectrometry SELDI - TOF MS: Surface-enhanced laser desorption/ionization Time-of-Flight mass spectrometry.

[2]. No. of original articles.

Table S17. Proteomics methodological approaches and technologies in original articles with sheep or goat work and proteomics, in accord with the tissue analysed.

	Tissue analysed																												
Methodological approaches and technologies [1]	no tissue	abomasum	adipose tissue	blood	bone	brain	cerebrospinal fluid	colostrum	embryo	fibre	heart	intestine	liver	lung	mammary gland	milk fat globule membrane	milk	muscle	ovary	oviduct	rumen	saliva	semen	skin	spermatozoa	spleen	testis	uterus	vaginal excretions
2-DE / SDS-PAGE	0	0	0	3	0	0	0	2	0	0	0	0	1	0	1	0	5	1	1	1	0	0	0	0	0	0	0	1	0
2-DE & LC-MS/MS	0	0	1	8	1	0	2	0	1	0	0	1	4	0	0	1	10	4	2	1	2	0	5	0	2	0	0	3	0
2-DE & MALDI-TOF MS	0	1	0	7	0	0	1	1	0	0	0	2	7	2	4	0	9	9	1	0	1	0	1	5	1	0	1	1	1
2-DE, MALDI-TOF MS, LC-MS/MS	0	2	0	0	0	0	0	0	0	0	1	0	0	0	0	3	2	0	0	0	0	1	0	0	0	0	0	0	1
in silico analysis	4	0	1	1	0	0	0	0	0	0	0	0	0	0	0	0	1	1	0	0	0	0	0	0	0	0	0	0	0
GeLC-MS/MS	0	2	0	3	0	1	1	1	1	1	0	0	2	1	1	7	11	3	3	0	2	1	5	2	2	1	1	0	2
LC-MS/MS	0	2	5	18	2	7	0	7	8	8	6	6	6	4	6	16	39	23	14	2	9	3	13	14	3	3	4	11	0
LC-MS/MS & MALDI-TOF MS	0	0	0	0	0	0	0	0	0	0	1	0	0	0	0	0	2	0	0	0	0	1	0	0	0	0	0	0	0
MALDI / SELDI - TOF MS	0	0	0	3	2	1	0	0	0	0	0	0	0	0	0	0	2	0	0	0	0	0	0	0	0	0	0	0	0
Protein microarrays	0	0	0	1	0	0	0	0	0	0	0	0	0	0	0	0	0	0	0	0	0	0	0	0	0	0	0	0	0

SDS-PAGE & MALDI-TOF MS	0	0	0	2	0	0	0	0	0	0	0	0	0	0	0	2	0	0	0	0	0	2	0	1	0	0	0	0	0

[1] 2-DE: two-dimensional gel electrophoresis, SDS-PAGE: Sodium dodecyl-sulfate polyacrylamide gel electrophoresis, LC-MS/MS: liquid chromatography-tandem mass spectrometry, MALDI-TOF MS: matrix-assisted laser desorption/ionization coupled to time-of-flight mass spectrometry, GeLC-MS/MS: polyacrylamide gel electrophoresis followed by liquid chromatography-tandem mass spectrometry SELDI - TOF MS: Surface-enhanced laser desorption/ionization Time-of-Flight mass spectrometry.

[2] No. of original articles

Table S18. Keywords featured in > 3 published papers with sheep or goat work and proteomics.

Keyword	No. of published papers, in which it appeared
proteomics	135
sheep	80
proteome	46
goat	39
mass spectrometry	29
2-DE	28
ovine	20
isobaric tag for relative and absolute quantitation (iTRAQ)	19
biomarker	18
proteomic	18
goat milk	16
milk	15
mastitis	14
colostrum	13
protein	13
metabolomics	12
whey proteins	11
MFGM proteins	10
LC-MS/MS	9
tandem mass tag	9
data-dependent acquisition	8
ewe	8
lactation	8
meat quality	8
milk protein	8
RNA-sequencing	8
transcriptome	8
2-D DIGE	7
label-free proteomics	7
mature milk	7
ovary	7
ruminant	7
seminal plasma	7
skeletal muscle	7
transcriptomics	7
Cashmere goat	6
milk fat globule membrane	6
proteomic analysis	6
quantitative proteomics	6
saliva	6
uterus	6
animal model	5
bioinformatics	5
cerebrospinal fluid	5
diagnosis	5
fertility	5
genomics	5
hair follicle	5
human	5
hypothalamus	5
keratin	5
liver	5
MALDI-ToF MS	5
mammary gland	5
metabolism	5

nutrition	5
Ovis aries	5
reproduction	5
rumen	5
SDS-PAGE	5
sperm	5
spermatozoa	5
subclinical mastitis	5
Tibetan sheep	5
vaccine	5
adaptation	4
animal proteomics	4
bioactive peptides	4
bovine milk	4
Capra hircus	4
caprine	4
clustering	4
dairy goat	4
early pregnancy	4
Echinococcus granulosus	4
endometrium	4
enzyme	4
Fasciola hepatica	4
HPLC	4
inflammation	4
label-free	4
label-free quantification	4
melatonin	4
microarray	4
multi-omics	4
phosphoproteome	4
phosphorylation	4
pregnancy	4
prolificacy	4
ram	4
seasonal weight loss	4
semen	4
serum	4
shotgun proteomics	4
Small Tail Han sheep	4
somatic cell counts	4
UHPLC-Q-Orbitrap HRMS	4
Western blot	4

Figure S7. Word cloud of the most frequently occurring keywords in published papers with sheep or goat work and proteomics.

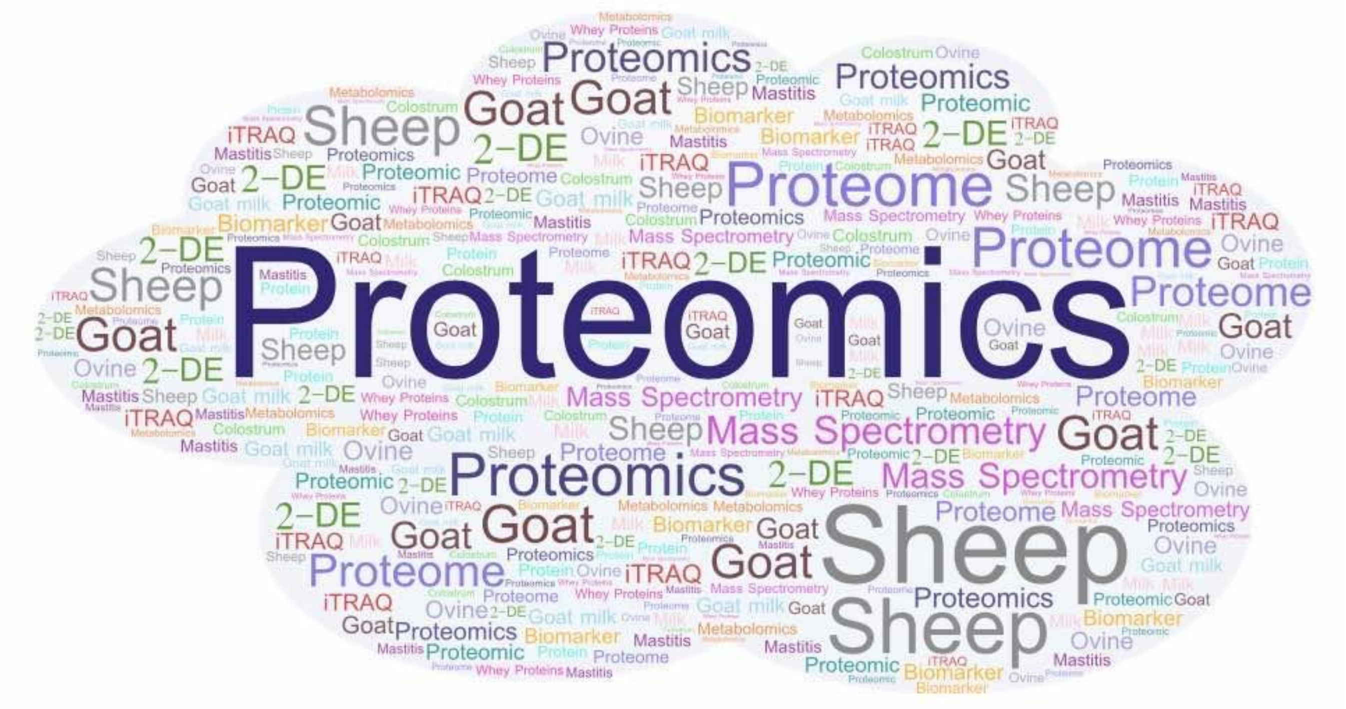

Figure S8. Scatter plot of the number of keywords per published papers with sheep or goat work and proteomics in accord with year of publication (dashed line is trendline).

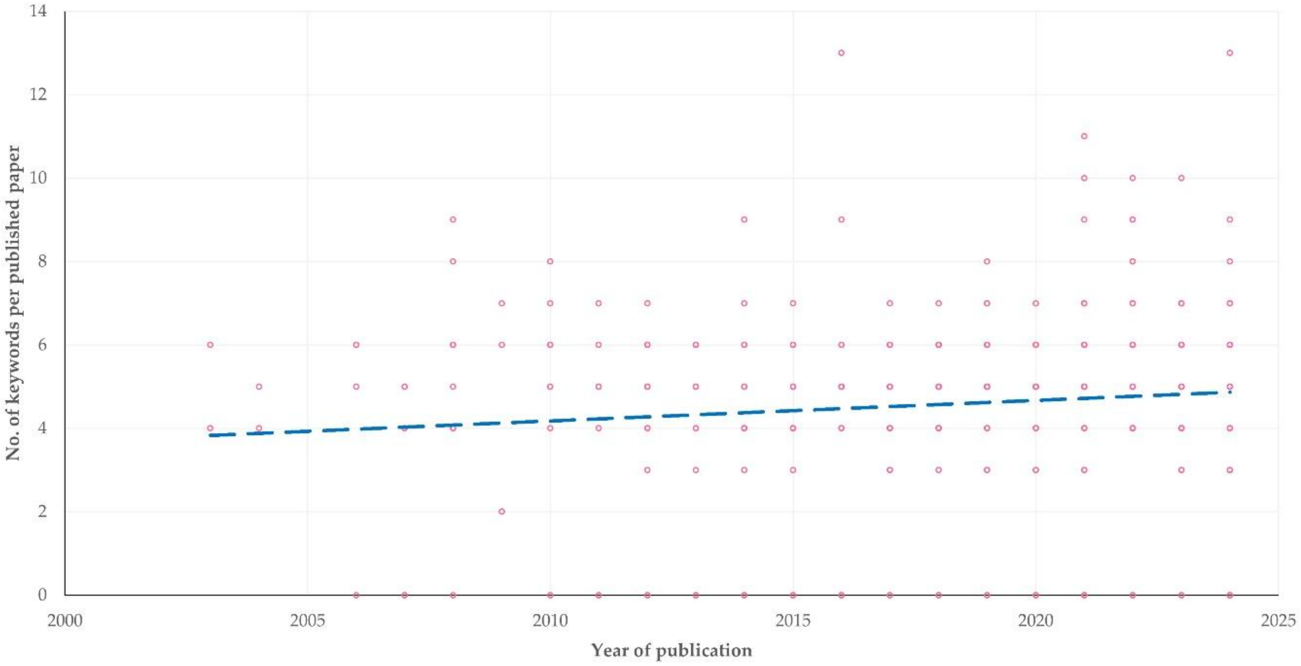

Table S19. Journals with published papers with sheep or goat work and proteomics.

Journal	No. of published papers
Journal of Proteomics	40
Animals	20
International Journal of Molecular Sciences	16
Journal of Dairy Science	15
Food Chemistry	14
Proteomics	14
PLOS One	11
Scientific Reports	11
Frontiers in Veterinary Science	10
Small Ruminant Research	9
International Dairy Journal	8
BMC Genomics	7
Electrophoresis	6
Foods	6
Journal of Dairy Research	6
Journal of Proteome Research	6
Reproduction in Domestic Animals	6
Research in Veterinary Science	6
Animal Reproduction Science	5
Journal of Agricultural and Food Chemistry	5
Journal of Animal Science and Biotechnology	5
Meat Science	5
Frontiers in Genetics	4
International Journal of Biological Macromolecules	4
Journal of Animal Science	4
Pathogens	4
Proteome Science	4
Reproduction	4
Veterinary Microbiology	4
Veterinary Research	4
Biology of Reproduction	3
Biology - Basel	3
Data in Brief	3
Food Chemistry - X	3
Food Research International	3
Frontiers in Immunology	3
Genes	3
Indian Journal of Animal Research	3
International Journal for Parasitology	3
Journal of Integrative Agriculture	3
Nutrients	3
Parasite Immunology	3
PeerJ	3
Veterinary Parasitology	3
Animal Genetics	2
Animal Nutrition	2
Animal Reproduction	2
Biochemical and Biophysical Research Communications	2
BMC Veterinary Research	2
Comparative Immunology Microbiology and Infectious Diseases	2
Frontiers in Animal Science	2
Frontiers in Cell and Developmental Biology	2
Frontiers in Physiology	2
Genetics and Molecular Research	2
Infection and Immunity	2

Italian Journal of Animal Science	2
Journal of Animal Physiology And Animal Nutrition	2
Journal of Biological Chemistry	2
Journal of Microbiological Methods	2
Kafkas Universitesi Veteriner Fakultesi Dergisi	2
Molecular Biosystems	2
Parasitology	2
Proteomics Clinical Applications	2
Tropical Animal Health and Production	2
Vaccines	2
Veterinary Journal	2
Acta Tropica	1
Acta Virologica	1
All Life	1
American Journal of Physiology - Endocrinology and Metabolism	1
American Journal of Physiology - Gastrointestinal and Liver Physiology	1
American Journal of Physiology - Lung Cellular and Molecular Physiology	1
Analytical and Bioanalytical Chemistry	1
Analytical Biochemistry	1
Analytical Chemistry	1
Animal	1
Animal Bioscience	1
Animal Biotechnology	1
Animal Feed Science and Technology	1
Animal Production Science	1
Asian - Australasian Journal of Animal Sciences	1
Austral Journal of Veterinary Sciences	1
Australian Journal of Agricultural Research	1
Biochimica et Biophysica Acta - Molecular Basis of Disease	1
Biomedicines	1
Biopreservation and Biobanking	1
Bioscience Reports	1
BMC Genetics	1
BMC Microbiology	1
BMC Research Notes	1
Breastfeeding Medicine	1
British Journal of Nutrition	1
Bulletin of the Veterinary Institute in Pulawy	1
Cardiovascular Research	1
Cells	1
Chinese Journal of Analytical Chemistry	1
Communications Biology	1
Comparative Biochemistry and Physiology D - Genomics & Proteomics	1
Critical Reviews in Food Science and Nutrition	1
Cryobiology	1
Current Protein & Peptide Science	1
Current Proteomics	1
Czech Journal of Animal Science	1
Dairy Science & Technology	1
Disease Models & Mechanisms	1
Domestic Animal Endocrinology	1
Egyptian Journal of Chemistry	1
Electronic Journal of Biotechnology	1
Emirates Journal of Food and Agriculture	1
Environment International	1
Environmental Science and Pollution Research	1
European Food Research and Technology	1

European Journal of Obstetrics & Gynecology and Reproductive Biology	1
Experimental Biology and Medicine	1
Experimental Eye Research	1
Experimental Gerontology	1
Experimental Parasitology	1
Fibers	1
Food & Function	1
Food Analytical Methods	1
Food Bioscience	1
Food Science and Biotechnology	1
Food Science and Technology	1
Frontiers in Endocrinology	1
Frontiers in Microbiology	1
Frontiers in Nutrition	1
Functional & Integrative Genomics	1
Gene	1
Genomics	1
Growth Hormone & IGF Research	1
Immunogenetics	1
Indian Journal of Animal Sciences	1
Indian Journal of Biochemistry & Biophysics	1
International Immunopharmacology	1
International Journal of Biometeorology	1
International Journal of Clinical and Experimental Pathology	1
International Journal of Food Science and Technology	1
Investigative Ophthalmology & Visual Science	1
IScience	1
ISME Journal	1
Journal of Archaeological Science	1
Journal of Chemical Ecology	1
Journal of Elementology	1
Journal of Endocrinology	1
Journal of Food Composition and Analysis	1
Journal of Food Measurement and Characterization	1
Journal of Food Science	1
Journal of Functional Foods	1
Journal of Huntington's Disease	1
Journal of Ovarian Research	1
Journal of Physiology - London	1
Journal of Reproduction and Development	1
Journal of The Science of Food and Agriculture	1
Journal of Toxicology and Environmental Health - Part A - Current Issues	1
Livestock Science	1
LWT - Food Science and Technology	1
Microbial Pathogenesis	1
Microcirculation	1
Mljekarstvo	1
Molecular & Cellular Proteomics	1
Molecular and Biochemical Parasitology	1
Molecular and Cellular Endocrinology	1
Molecular Biology and Evolution	1
Molecular Biology Reports	1
Molecular Human Reproduction	1
Molecular Immunology	1
Molecular Medicine	1
Molecular Reproduction and Development	1
Molecules	1

Neural Regeneration Research	1
Neuroscience Letters	1
Peptides	1
Pharmaceutical Biology	1
Physiological Genomics	1
Physiology & Behavior	1
PLOS Neglected Tropical Diseases	1
Polish Journal of Veterinary Sciences	1
Progress in Biochemistry and Biophysics	1
Proteins - Structure Function and Bioinformatics	1
Rapid Communications in Mass Spectrometry	1
Reproductive Toxicology	1
Revista Brasileira de Zootecnia - Brazilian Journal of Animal Science	1
Science of the Total Environment	1
Science Translational Medicine	1
Scientia Agricola	1
Scientific Data	1
Stem Cells and Development	1
Theriogenology	1
Thrombosis Research	1
Toxicology Research	1
Tropical Biomedicine	1
Turkish Journal of Veterinary & Animal Sciences	1
Ultrasonics Sonochemistry	1
Veterinary Immunology and Immunopathology	1
Veterinary Quarterly	1
Veterinary Sciences	1
Waste And Biomass Valorization	1
Zeitschrift fur Naturforschung Section C - A Journal of Biosciences	1

Figure S9. Box-and-whisker plot of year of publication of papers in the five journals with most papers and content on proteomics – molecular studies [1] (grey plot; n = 82)) and in the eight journals with most papers and content on clinical – health management studies [2] (green plot; n = 77)).

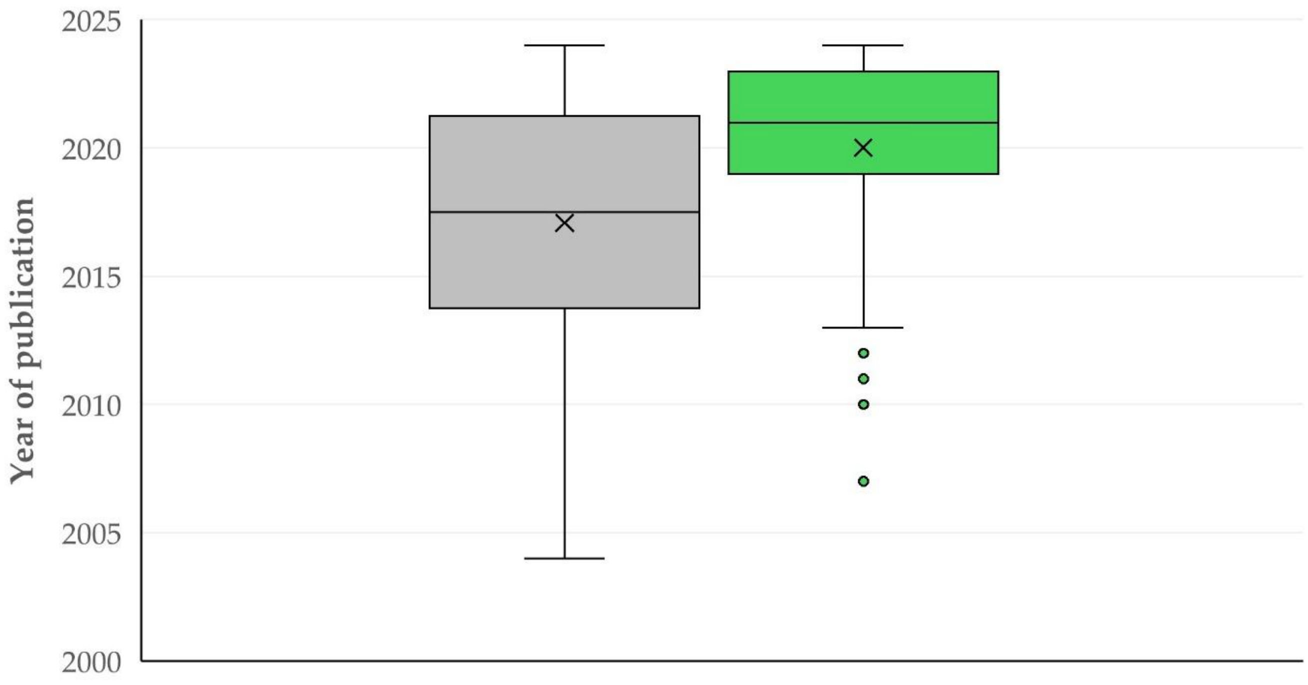

[1] *Journal of Proteomics, International Journal of Molecular Sciences, Proteomics, Electrophoresis, Journal of Proteome Research*
[2] *Animals, Journal of Dairy Science, Frontiers in Veterinary Science, Small Ruminant Research , Journal of Dairy Research, Reproduction in Domestic Animals, Research in Veterinary Science, Animal Reproduction Science*.

Figure S10. Scatter plot of the number of authors per published papers with sheep or goat work and proteomics in accord with year of publication (dashed line is trendline).

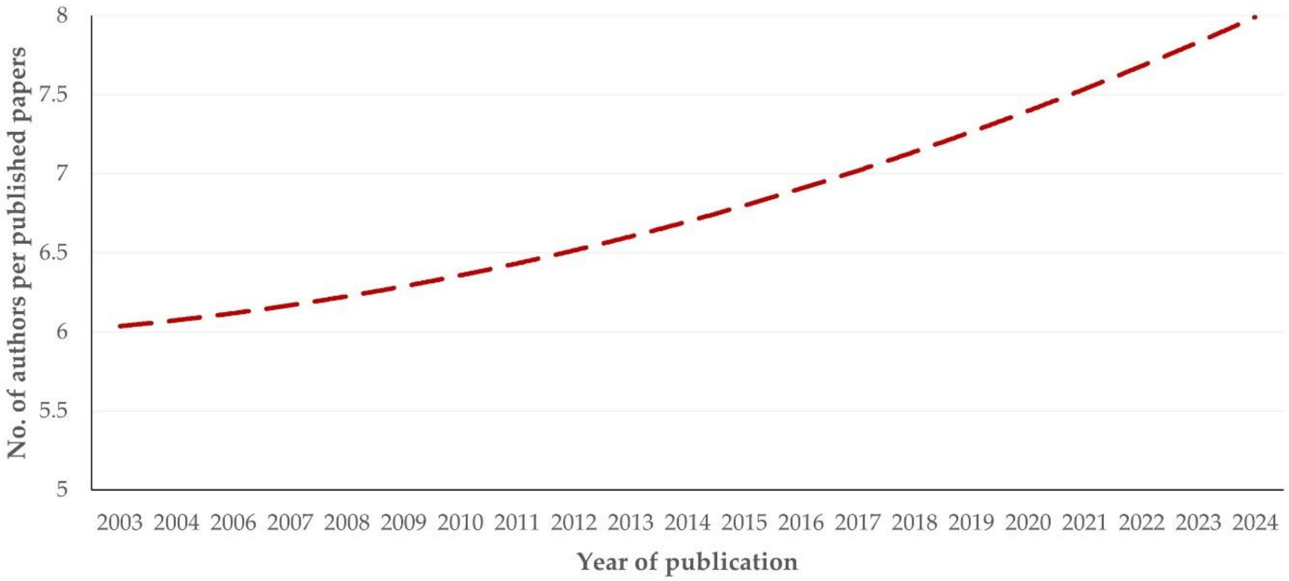

Table S20. Number of authors in published papers with sheep or goat work and proteomics, in accord with the country of origin [1].

Country	Median no. of authors per published paper
Australia	8 (IQR: 5)
Brazil	11 (IQR: 4)
China	8 (IQR: 4)
France	8 (IQR: 6
Germany	8 (IQR: 3.5)
Greece	8 (IQR: 3.5)
India	6.5 (IQR: 6)
Italy	8 (IQR: 4)
New Zealand	8 (IQR: 3)
Portugal	10 (IQR: 5)
Spain	9 (IQR: 3)
United Kingdom	6 (IQR) 4)
United States of America	6.5 (IQR: 8)

[1] Only countries with most (> 10) published papers considered.

Table S21. Number of authors in original articles with sheep or goat work and proteomics, in accord with the topic of study.

Topic of study	Median no. of authors per published paper
Animal diseases	8 (IQR: 4)
Use of small ruminants as models	10 (IQR: 7)
Physiology	8 (IQR: 5)
Sheep / goat production	7 (IQR) 4)
Sheep / goat reproduction	8 (IQR: 5)

Table S22. Number of cited references in original articles with sheep or goat work and proteomics, in accord with the type of work.

Type of work	Median no. of cited references per original article
Field	50 (IQR: 29.5)
Computational	59 (IQR: 20)
Experimental	48 (IQR: 27)
In vitro	49 (IQR) 11)

Table S23. Number of cited references in original articles with sheep or goat work and proteomics, in accord with the topic of study.

Topic of study	Median no. of authors per published paper
Animal diseases	49.5 (IQR: 37)
Use of small ruminants as models	60 (IQR: 33.5)
Physiology	49 (IQR: 28)
Sheep / goat production	41 (IQR: 17.5)
Sheep / goat reproduction	56.5 (IQR: 29)

Table S24. Results of univariable analyses for predictors associated with the number of annual citations in original articles with sheep or goat work and proteomics.

Variable		*p*
Animal species referred to in paper		0.010
Sheep	1.6 (2.7) [1]	
Goats	2.0 (3.1)	
Both	2.6 (2.0)	
Year of publication		0.39
	r_{sp} = −0.041 [2]	
No. of countries of origin		0.39
	r_{sp} = 0.041	
No. of establishments of affiliation		0.87
	r_{sp} = −0.008	
Country of first author		0.09
Algeria	12.0 (3.3)	
Australia	1.7 (2.8)	
Austria	1.6 (2.0)	
Brasil	1.2 (2.2)	
Canada	1.6 (0.0)	
China	2.0 (3.1)	
Denmark	1.3 (0.0)	
Egypt	0.8 (0.0)	
Finland	1.0 (0.0)	
France	1.7 (1.9)	
Germany	2.0 (1.5)	
Greece	0.0 (2.0)	
Hungary	0.5 (0.0)	
India	0.8 (3.5)	
Indonesia	0.1 (0.0)	
Iran	1.5 (0.8)	
Ireland	6.4 (8.7)	
Italy	1.5 (1.8)	
Japan	1.4 (0.0)	
Malaysia	2.2 (0.6)	
Mexico	2.0 (0.0)	
New Zealand	1.1 (2.1)	
Norway	8.2 (7.1)	
Poland	0.4 (0.2)	
Portugal	2.5 (1.4)	
Saudi Arabia	1.7 (0.0)	
Slovakia	0.6 (0.0)	
South Africa	0.1 (0.0)	
Spain	2.6 (2.3)	
Saint Kitts	2.2 (0.9)	
Switzerland	2.1 (1.2)	
Taiwan	1.6 (0.0)	
Tunisia	7.8 (0.0)	
Turkiye	6.0 (0.0)	
United Kingdom	2.2 (2.8)	
United States of America	2.7 (4.0)	
Venezuela	0.4 (0.0)	
Type of work		0.58
Field	1.9 (3.7)	
Experimental	2.0 (2.9)	
Computational	1.2 (5.3)	
In vitro	1.4 (1.5)	

Topic of study		0.008
Animal diseases	1.5 (2.0)	
Use of small ruminants as models	1.7 (2.2)	
Physiology	1.7 (3.1)	
Sheep / goat production	2.5 (3.0)	
Sheep / goat reproduction	1.8 (2.9)	
Proteomics methodological approaches and technologies used		0.11
2-DE / SDS-PAGE	2.0 (2.0)	
2-DE & LC-MS/MS	1.7 (2.1)	
2-DE & MALDI-TOF MS	1.3 (1.9)	
2-DE & MALDI-TOF MS & LC-MS/MS	1.9 (1.7)	
in silico analysis	1.1 (2.0)	
GeLC-MS/MS	2.8 (3.3)	
LC-MS/MS	2.0 (3.2)	
LC-MS/MS & MALDI-TOF MS	1.6 (0.3)	
MALDI / SELDI - TOF MS	1.7 (1.4)	
Protein microarrays	2.1 (0.7)	
SDS-PAGE & MALDI-TOF MS	1.8 (1.6)	
Quantification analysis		0.66
With quantification analysis	2.0 (3.1)	
Without quantification analysis	2.0 (2.9)	
Use of additional -omics technologies		0.21
With use of additional -omics technologies	2.0 (2.8)	
Without use of additional -omics technologies	2.0 (2.8)	
No. of keywords		0.29
	$r_{sp} = -0.050$	
Number of references cited in the paper		0.0001
	$r_{sp} = 0.178$	
Accessibility of paper		0.53
Open access	2.0 (2.9)	
No open access	1.8 (2.9)	

[1] Median value (interquartile range).

[2] Spearman correlation coefficient.

Table S25. Results of univariable analyses for predictors associated with the number of annual citations in reviews with sheep or goat work and proteomics.

Variable		*p*
Animal species referred to in paper		0.67
Sheep	1.6 (4.6)	
Goats	2.1 (3.9)	
Both	3.3 (5.3)	
Year of publication		0.36
	r_{sp} = 0.166 [2]	
No. of countries of origin		0.74
	r_{sp} = −0.062	
No. of establishments of affiliation		0.94
	r_{sp} = 0.014	
Country of first author		0.87
Albania	0.5 (0.0)	
Australia	2.2 (4.4)	
China	4.7 (3.5)	
Czechia	0.5 (7.8)	
France	10.6 (0.0)	
Germany	2.7 (0.0)	
Greece	1.5 (2.2)	
India	2.2 (1.1)	
Israel	8.2 (0.0)	
Italy	6.0 (3.1)	
Malaysia	1.2 (0.0)	
New Zealand	1.8 (0.0)	
Poland	1.6 (1.6)	
United Kingdom	3.8 (4.8)	
United States of America	1.9 (0.3)	
No. of keywords		0.40
	r_{sp} = −0.150	
Number of references cited in the paper		0.007
	r_{sp} = 0.462	
Accessibility of paper		0.86
Open access	2.7 (4.9)	
No open access	1.7 (4.5)	

[1] Median value (interquartile range).

[2] Spearman correlation coefficient.

Figure S11. Scree plot for principal component analysis for the number of annual citations in original articles with sheep or goat work and proteomics.

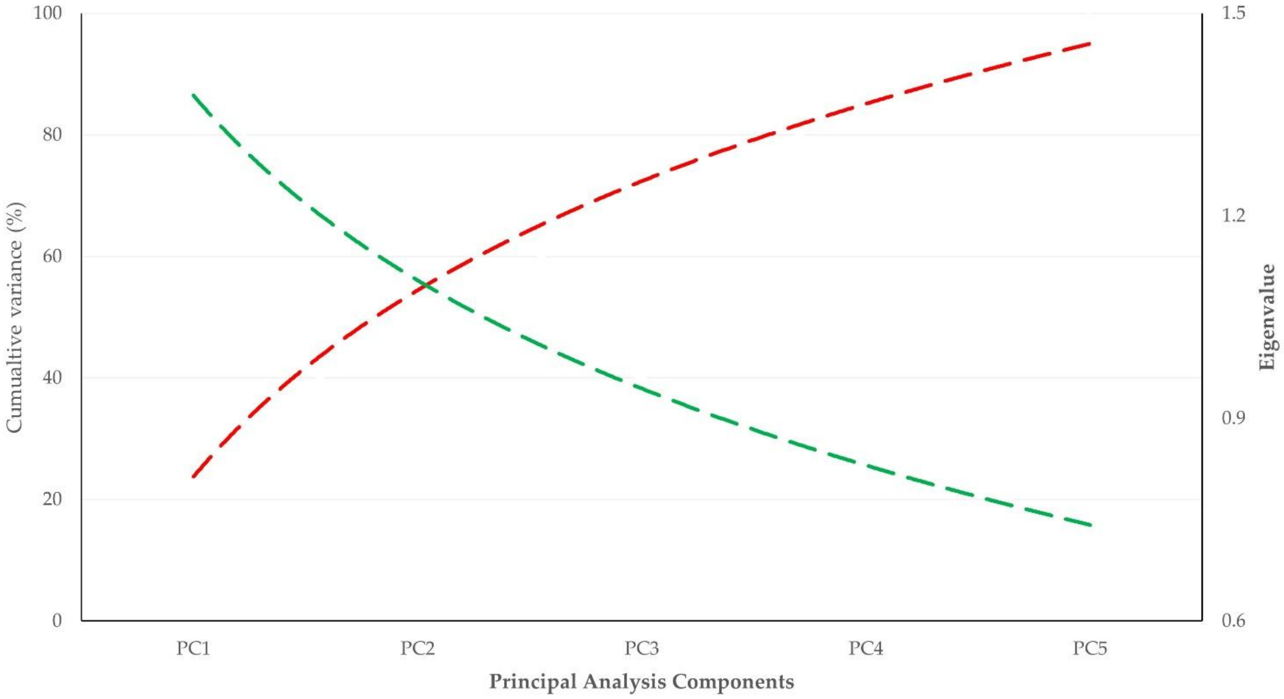

Supplement: Supplementary file 1 [file animals-15-03050-s001.zip › animals-3892649-supplementary.pdf]
